# Decoupled neural network training with re-computation and weight prediction

**DOI:** 10.1371/journal.pone.0276427

**Published:** 2023-02-23

**Authors:** Jiawei Peng, Yicheng Xu, Zhiping Lin, Zhenyu Weng, Zishuo Yang, Huiping Zhuang

**Affiliations:** 1 School of Electrical and Electronic Engineering, Nanyang Technological University, Singapore, Singapore; 2 Shien-Ming Wu School of Intelligent Engineering, South China University of Technology, Guangzhou, China; Sichuan University, CHINA

## Abstract

To break the three lockings during backpropagation (BP) process for neural network training, multiple decoupled learning methods have been investigated recently. These methods either lead to significant drop in accuracy performance or suffer from dramatic increase in memory usage. In this paper, a new form of decoupled learning, named decoupled neural network training scheme with re-computation and weight prediction (DTRP) is proposed. In DTRP, a re-computation scheme is adopted to solve the memory explosion problem, and a weight prediction scheme is proposed to deal with the weight delay caused by re-computation. Additionally, a batch compensation scheme is developed, allowing the proposed DTRP to run faster. Theoretical analysis shows that DTRP is guaranteed to converge to crical points under certain conditions. Experiments are conducted by training various convolutional neural networks on several classification datasets, showing comparable or better results than the state-of-the-art methods and BP. These experiments also reveal that adopting the proposed method, the memory explosion problem is effectively solved, and a significant acceleration is achieved.

## 1 Introduction

Deep learning has been widely applied in various fields, such as the medical [1–3] and the robotics [4–6] fields. Through significant breakthroughs in the areas, neural networks, such as ResNet [7] and DenseNet [8], have been designed to handle very deep network structure. This allows the model to achieve more robust performance for deployment, providing real-life business solutions. It has been shown both in theory [9, 10] and practice [7, 8, 11–13] that depth is one of the most important factors leading to the success of deep learning. However, the bottleneck lies in the training of deep neural network which is computationally heavy and time-consuming.

A potential solution can be directed to improving the common training mechanism of networks, namely the back-propagation (BP) [14]. A standard BP consists of the forward pass, backward pass and parameter update. The input is passed to the neural network forwardly layer by layer and the gradients also flow back layer by layer. New training iteration can only start after all the parameters in the network are updated. Thus, each layer of the network is locked until their dependent parameters have been computed. These are known as forward, backward and update lockings [15], which cause inefficiency [16] since the majority of the network stays idle during the training process. As deeper neural networks are developed nowadays, the inefficiency that occurs in the standard BP becomes more serious.

To address such inefficiency, *decoupled learning*, a form of model parallelism, has been developed to improve the efficiency of the training process in recent years. This technique generally divides the network into several modules (each module contains a consecutive number of layers) and trains them on different processors in parallel. The parallel computing of the decoupled model improves the training efficiency significantly. Ref. [17] introduced a decoupled parallel BP algorithm with delayed gradient (DDG) that breaks the backward locking using delayed gradients while maintaining a high performance of very deep networks. A following feature replay method (FR) [18] also breaks the backward locking by introducing re-computation of input features. It even outperforms the BP on several deep convolutional neural network (CNN) models. The FR scheme reduces the memory consumption but involves a larger amount of computation. These backward-unlocking based methods did not fully break all the lockings and the acceleration is constrained.

Designing auxiliary networks for local learning solves the locking problem from another angle. In the decoupled neural interface (DNI) [15], local neural networks are built to produce local error gradients through synthesizing gradients so that earlier layers can perform the update before completing the backward pass. Hence, modules can be trained asynchronously in parallel. This is a pioneering work that is promising to apply to the parallel training of neural networks. A follow-up method utilizes local classifier with cross-entropy loss to produce local gradients [19]. However, the performance of local error based methods gets far worse when models become deeper. Recently, a decoupled greedy learning (DGL) [20] method has been proposed which shows acceptable results for very deep networks, but it introduces extra trainable parameters due to the auxiliary networks. Furthermore, the performance of these methods using local error learning is highly dependent on the design of auxiliary networks.

The fully decoupled neural network learning using delayed gradients (FDG) [21] can break all the lockings and achieve model parallelization without the introduction of extra trainable parameters. FDG could achieve significant acceleration. However, the memory explosion problem emerges especially when the number of split modules increases. Moreover, the imbalanced split of the modules causes each module to have different computation time at every iteration. The overall speed of training is encumbered by the slowest module, hence limiting the acceleration of training. A recently proposed accumulated decoupled learning (ADL) [22] carries the same memory limitation.

This has motivated us to develop a new decoupled learning technique which can address all the locking issues while having comparable results to those of BP, and without being constrained by the memory problem. In this paper, a decoupled network training with re-computation and weight prediction (DTRP) is proposed. The main contributions of this work can be summarized as follows:

- We propose the DTRP, which adopts a re-computation technique that addresses the issues of memory explosion, and a weight prediction scheme to mitigate the effect of weight staleness caused by the re-computation scheme. Such a new decoupled learning method can break all three lockings in BP.
- A pioneering batch compensation method is explored to reduce the effect on imbalanced model splitting to accelerate the training. To the best of our knowledge, the imbalanced issue in decoupled learning has not been explored in previous works.
- We provide a theoretical analysis to prove that our proposed DTRP method is guaranteed to converge statistically under certain conditions.
- Our empirical results show that the DTRP provides comparable or better results against state-of-the-art decoupled learning methods as well as the standard BP in terms of generalization ability. We also show by experiments that the DTRP solves the memory explosion effectively and achieves significant acceleration.

The remaining part of the paper is summarized as follows. Section 2 provides the background knowledge to develop the DTRP. Section 3 introduces the proposed DTRP in detail. Section 4 introduces three theorems to conduct the convergence analysis of the DTRP. Section 5 describes the details of experiments and presents the results. The conclusion is drawn in Section 6. Finally, proofs of three theorems in convergence analysis of the DTRP are provided in the S1 File.

## 2 Background

In this section, some background knowledge of training feed-forward neural network is provided. Also, the problem of forward, backward and update lockings is illustrated [15].

Consider a deep neural network with L layers. For the *l*^th^ layer (1≤l≤L), it can only receive the output from the previous layer to perform computation and pass the output as the input to the next layer. Hence the output can be expressed as

$$\begin{array}{c} a_{l}=A_{l}\left(a_{l-1},w_{l}\right) \end{array}$$ (1)

where ***a***_*l*−1_ is the input of layer *l*, which is also the activation from layer *l* − 1 and ***w***_*l*_ represents the weights in layer *l*. The output of layer *l* cannot be computed until the output of layer *l* − 1 is obtained. Hence, the feed-forward process, which requires each layer to wait for the dependent activations of the previous layer, causes *forward locking* [15]. This is not efficient as at any time, only one layer is performing computations while others are idle and waiting for the dependent input.

To train a neural network, a loss function needs to be defined to evaluate how well it predicts the output based on the input. The goal of training is to minimize the loss function so that the network can predict the output as accurately as possible. Assume *L* is the loss function mapping the weights to a scalar loss value and *L*_*x*_ represents the loss function calculated using a batch of training samples *x*. The objective function of training a neural network can be summarized as

$$\begin{array}{c} \underset{w=\{w_{1},w_{2},\ldots ,w_{l}\}}{\text{minimize}}\;L_{x}\left(w_{1},w_{2},\ldots ,w_{l}\right). \end{array}$$ (2)

To update parameters ***w***, gradient descent [23] is usually adopted to calculate the gradient that implies the direction of optimization. At iteration *t*, for layer *l*, the gradient can be obtained by

$$\begin{array}{c} g_{w_{l}}^{t}=\frac{\partial L_{x}\left(w^{t}\right)}{\partial w_{l}^{t}}. \end{array}$$ (3)

By applying chain rule, the error at the output layer can be propagated back to the hidden layer [14]. For layer *l*, the gradient can be calculated from layer *l* + 1 as shown below:

$$\begin{array}{c} g_{w_{l}}^{t}=\frac{\partial L_{x}\left(w^{t}\right)}{\partial w_{l}^{t}}=\frac{\partial L_{x}\left(w^{t}\right)}{\partial a_{l}^{t}}\frac{\partial a_{l}^{t}}{\partial w_{l}^{t}}=g_{a_{l}}^{t}\frac{\partial a_{l}^{t}}{\partial w_{l}^{t}} \end{array}$$ (4)

$$\begin{array}{c} g_{a_{l}}^{t}=\frac{\partial L_{x}\left(w^{t}\right)}{\partial a_{l}^{t}}=\frac{\partial L_{x}\left(w^{t}\right)}{\partial a_{l+1}^{t}}\frac{\partial a_{l+1}^{t}}{\partial a_{l}^{t}}=g_{a_{l+1}}^{t}\frac{\partial a_{l+1}^{t}}{\partial a_{l}^{t}}. \end{array}$$ (5)

Eqs (4) and (5) indicate that the gradient of layer *l* is dependent on the gradient of next layer *l* + 1. The gradient can only be calculated until the dependent gradients flow back to layer *l*. This is known as *backward locking* [15]. This causes inefficiency during the backward pass of the training.

After obtaining all the gradients, one can update the weights through the gradient descent algorithm as follows:

$$\begin{array}{c} w_{l}^{t+1}=w_{l}^{t}-\eta_{t}g_{w_{l}}^{t}. \end{array}$$ (6)

The learning rate *η*_*t*_ determines the steps taken to reach a smaller loss. All layers are constrained for updating parameters before the forward pass is completed and the dependent gradients are obtained, which is referred to as *update locking*. The whole training process faces the issue of three lockings mentioned. Moreover, as the neural network grows deeper to solve more complicated problems, there are more trainable parameters and it is therefore computationally more intensive and more time-consuming, which amplifies the inefficiency of the lockings. Hence, it is of vital importance to break the lockings and leverage the computation resources efficiently.

## 3 Decoupled training with re-computation and weight prediction

This section presents the proposed decoupled network training with re-computation and weight prediction (DTRP) method in detail. We start with the decoupled training scheme with re-computation (DTR), and then introduce the complete DTRP that incorporates the weight prediction to alleviate the effect caused by stale weights in the DTR. In addition, a batch compensation is utilized to offset the waiting time and computing resource caused by imbalanced splitting of the model. In the end, we provide a comprehensive comparison with previous decoupled training method FDG.

### 3.1 Decoupled training with re-computation

**Fig 1:**
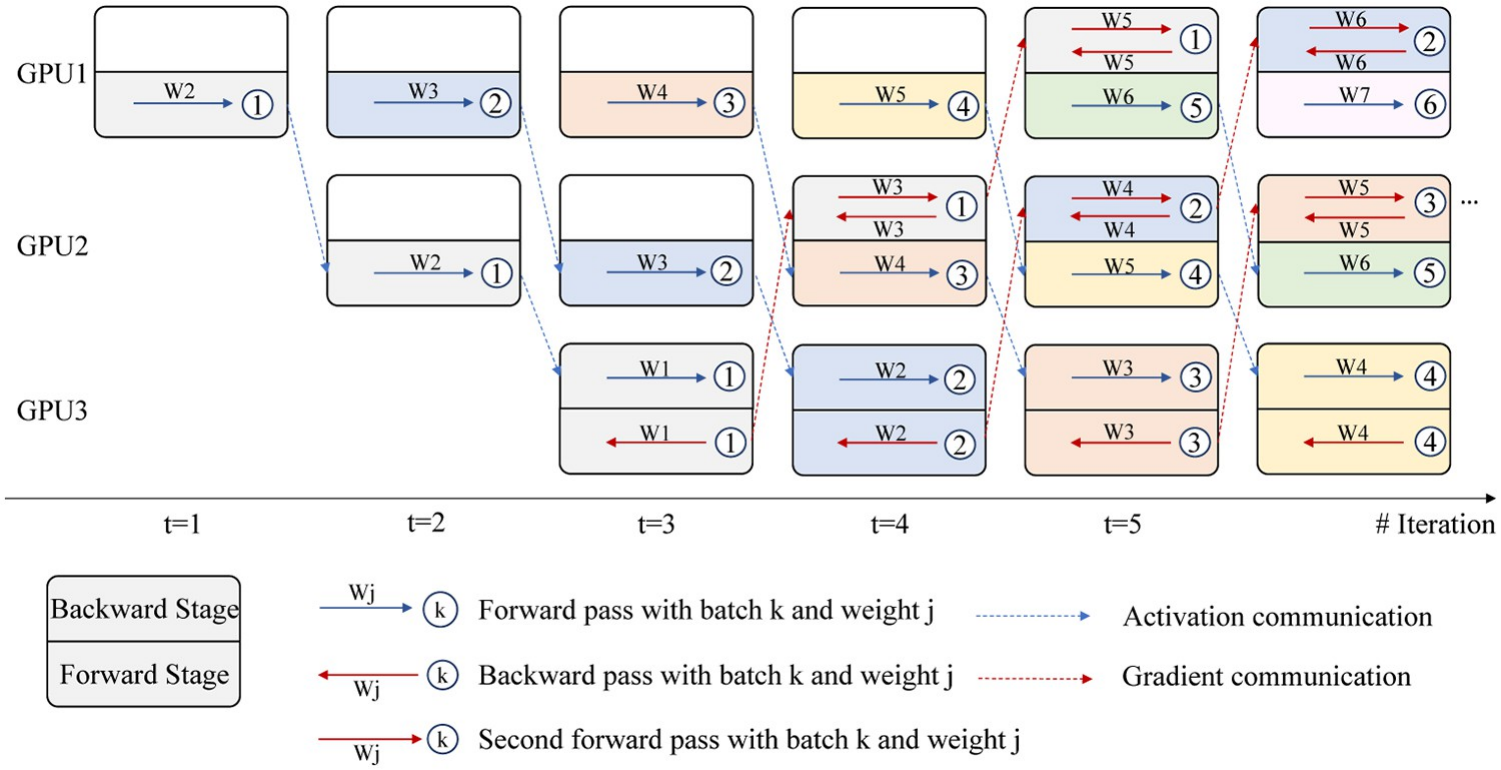
Illustration of Decoupled Training with Re-computation (DTR): A *K* = 3 example. Each color in the figure represents a completed forward and backward process in the whole model of a single batch.All modules execute backward stage (upper part) first and then forward stage (lower part) except the last module which processes reversely. In initial iterations where modules do not have backward stages, *w*^*t*−*k*+2^ is set to be equal to *w*^*t*−*k*+1^ (e.g. in module *m*_1_ at iteration *t* = 1, *W*2 = *W*1).

With a given partition number *K*, the network with L layers will be partitioned into *K* modules denoted by {*m*_1_, *m*_2_, …, *m*_*k*_, …, *m*_*K*_}. Each module *m*_*k*_ contains a sequence of consecutive layers $\left\{l_{k,1},l_{k,2},l_{k,3},\ldots ,l_{k,L_{k}}\right\}$ where *L*_*k*_ represents the number of layers in module *k*. Subsequently, they will be placed onto different processors for parallel training as shown in Fig 1.

• **Forward Stage**: In DTR, at iteration *t* of the forward stage, represented as the lower part of the box except for the last module, module *m*_*k*_ receives the activations from its previous module *m*_*k*−1_, and performs a forward pass (FP) to produce output. Such forward pass is referred to as the first forward pass (first FP) (1 < *k* < *K*):

$$\begin{array}{c} a_{l_{k-1,L_{k-1}}}^{t-k+1}\to m_{k}\left(w^{t-k+2}\right)\to a_{l_{k,L_{k}}}^{t-k+1}. \end{array}$$ (7)

The superscript of *t* − *k* + 1 for ***a*** represents the batch number. At iteration *t*, the delayed activations (a delay of *k* − 1) are used for the forward pass. *w* denotes the current weight of *m*_*k*_ used to produce activation. The superscript for *w* can be rewritten as *t* − *k* + 2 = *t* − (*k* − 1) + 1, where *k* − 1 is the delay of activation with respect to iteration *t*. The last term “+1” indicates that the forward stage is executed after backward stage so that module *m*_*k*_ uses parameters ***w***^*t*−*k*+2^ which have been updated in (11).

• **Backward Stage**: At the backward stage, denoted as the upper part of the box except for the last module, *m*_*k*_ receives gradients from the subsequent module *m*_*k*+1_. This backward stage consists of two operations, a forward pass for re-computation of activations using the same batch as the one which the gradient flowing back is based on, and a backward pass. Specifically, for the last module *m*_*K*_, the backward stage contains only a normal backward pass. This forward pass is referred to as the second forward pass (second FP):

$$\begin{array}{c} a_{l_{k-1,L_{k-1}}}^{t+k-2K+1}\to m_{k}\left(w^{t-k+1}\right)\to {\tilde{a}}_{l_{k,L_{k}}}^{t+k-2K+1}. \end{array}$$ (8)

The superscript *t* + *k* − 2*K* + 1 is used because a gradient delay of 2(*K* − *k*) is observed between the forward pass and gradient calculation with respect to batch *t* − *k* + 1. ${\tilde{a}}_{l_{k,L_{k}}}^{t+k-2K+1}$ indicates the activation generated in the second FP uses current weight ***w***^*t*−*k*+1^ while the actual activation $a_{l_{k,L_{k}}}^{t+k-2K+1}$ generated in the first FP at the previous iteration used weight ***w***^*t*+*k*−2*K*+2^.

After re-computing the activations obtained using batch *t* + *k* − 2*K* + 1, we can calculate the gradient for each layer *l* in $\left\{l_{k,1},l_{k,2},l_{k,3},\ldots ,l_{k,L_{k}}\right\}$ by

$$\begin{array}{c} {\hat{g}}_{w_{l}}^{t-k+1}={\hat{g}}_{a_{l_{k,L_{k}}}}^{t+k-2K+1}\frac{\partial {\tilde{a}}_{l_{k,L_{k}}}^{t+k-2K+1}}{\partial w_{l}^{t-k+1}} \end{array}$$ (9)

and the gradient ${\hat{g}}_{a_{l_{k-1,L_{k-1}}}}^{t+k-2K+1}$ w.r.t to the stored input of module *m*_*k*_ is calculated similarly as follows:

$$\begin{array}{c} {\hat{g}}_{a_{l_{k-1,L_{k-1}}}}^{t+k-2K+1}={\hat{g}}_{a_{l_{k,L_{k}}}}^{t+k-2K+1}\frac{\partial {\tilde{a}}_{l_{k,L_{k}}}^{t+k-2K+1}}{\partial a_{l_{k-1,L_{k}-1}}^{t+k-2K+1}} \end{array}$$ (10)

where ${\hat{g}}_{a_{l_{k,L_{k}}}}^{t+k-2K+1}$ is received from module *m*_*k*+1_ at the previous iteration t, so that all calculation of gradients in module *m*_*k*_ is independent of other modules. Furthermore, the new computational graph used for gradient calculation is generated by (8).

The gradient and the activations are calculated from the same batch of data. Subsequently, each layer in the module can be updated by

$$\begin{array}{c} w_{l}^{t-k+2}=w_{l}^{t-k+1}-\eta_{t}{\hat{g}}_{w_{l}}^{t-k+1}. \end{array}$$ (11)

It is worth pointing out that during the backward stage, re-computation is applied so that the computing devices do not need to store the activations and wait for the backward pass to be finished for the same batch to clear them. Without re-computation, all the activations in the computation graph that have not been used for gradients calculation have to be kept in memory for further gradients computation.

• **Communication**: After all the modules finish an iteration of forward and backward stage, the activations $a_{l_{k,L_{k}}}^{t-k+1}$ computed in *m*_*k*_ will be passed to *m*_*k*+1_ and the gradients obtained ${\hat{g}}_{a_{l_{k-1,L_{k-1}}}}^{t+k-2K+1}$ will be sent to *m*_*k*−1_.

Since delayed gradients are used to update the model parameters, this can lead to performance loss [24]. A learning rate shrinking (LRS) process in (12) is deployed to reduce the effect caused by stale gradients. We adopt a shrinking factor *γ* to scale the learning rate as follows:

$$\begin{array}{c} w_{l}^{t-k+2}=w_{l}^{t-k+1}-\gamma \eta_{t}{\hat{g}}_{w_{l}}^{t-k+1} \end{array}$$ (12)

where if *γ* = 1, the learning rate shrinking is not deployed.

Since all the modules perform each entire process asynchronously, they can be trained in parallel. As the backward stage is independent of the forward stage at each iteration because they are processing different batches of data, the backward stage is executed before the forward stage to reduce the delay in gradient. An exception is that the last module will perform the forward pass first, followed by a normal backward pass. That is the reason why two parts of the last module are reversal compared to other modules in Fig 1.

• **Analysis of Speedup**: The analysis of the model acceleration performance is shown in Table 1. The DTR fully breaks the backward locking and unlocks the forward locking. The backward pass usually costs more than twice of the computation time of the forward pass according to the benchmark report in [25]. Thus, re-computation of the forward pass does not introduce extra training time significantly while fully solving the main bottleneck—backward locking. It can be shown that the proposed method outperforms DDG [17] and FR [18] in terms of acceleration and is comparable to DGL [20], which is dependent on the computation load of auxiliary networks.

**Table 1:** Assume in the ideal case that the communication time between different GPUs is negligible and the model is evenly split into *K* modules. *T*_*f*_, *T*_*b*_ and *T*_*aux*_ denote the time taken for forward, backward passes, and the auxiliary network, respectively.

Methods	BP [14]	DDG [17]	FR [18]	DNI [15] & DGL [20]	FDG [21]	DTR (proposed)
Time	$T_{f}+T_{b}$	$T_{f}+\frac{T_{b}}{K}$	$T_{f}+\frac{T_{f}+T_{b}}{K}$	$\frac{T_{f}+T_{b}}{K}+T_{aux}$	$\frac{T_{f}+T_{b}}{K}$	$\frac{2T_{f}+T_{b}}{K}$

• **Analysis of Memory Usage**: Although FDG [21] can achieve the highest acceleration since it addresses all three lockings, it suffers from a memory explosion problem. The analysis of memory usage comparison between the FDG and the DTR is presented in Table 2. In the case without re-computation (i.e., FDG), all the input data and activation graphs generated need to be kept for further gradients computation. The activation graph takes significant space while the space taken by input and model parameters is relatively small compared with the activations. With the increasing of *K*, the delayed gradient effect is amplified, and the memory used by activation graphs increases significantly. This causes the memory to explode in the first several workers (e.g. GPUs). With the implementation of re-computation in DTR, the memory can be freed up by removing all the activation graphs since re-computation generates a new activation graph with the input stored in the memory.

**Table 2:** *M*_*input*_, *M*_*activations*_, and $M_{module_{k}}$ represent the memory space taken by one batch of input feature and activation graphs, and memory space taken by module *k* respectively.

Methods	Memory Usage
FDG	$\left[2\left(K-k\right)\right]M_{input}+\left[2\left(K-k\right)\right]M_{activations}+M_{module_{k}}$
DTR (proposed)	$\left[2\left(K-k\right)\right]M_{input}+M_{module_{k}}$

### 3.2 Weight prediction

The adoption of re-computation causes a difference between weights of modules used in the second FP during the backward stage and that used in the first FP during the forward stage for the same batch of data. Since the stale weights used for the first FP generate stale gradients from subsequent modules based on stale activations, this stale gradient vector does not correspond to the weights and activations produced during second FP. This may lead to a certain degree of performance loss. According to Section 3.1, at first FP, the index of weight is kept consistent with respect to the batch number (except that there is an update after the backward stage). At the second FP, the adopted weight does not correspond to the one of the first FP as it has been updated by being aligned with a new batch number. (i.e. with respect to batch *t* − 2*K* + *k* + 1, *w*^*t*−2*K*+*k*+2^ is used in the first FP while *w*^*t*−*k*+1^ is used in the second FP). The weight delay steps between two forward passes are shown as follows:

$$\begin{array}{c} \text{weight}\;\text{delay}=2(K-k)-1. \end{array}$$ (13)

**Fig 2:**
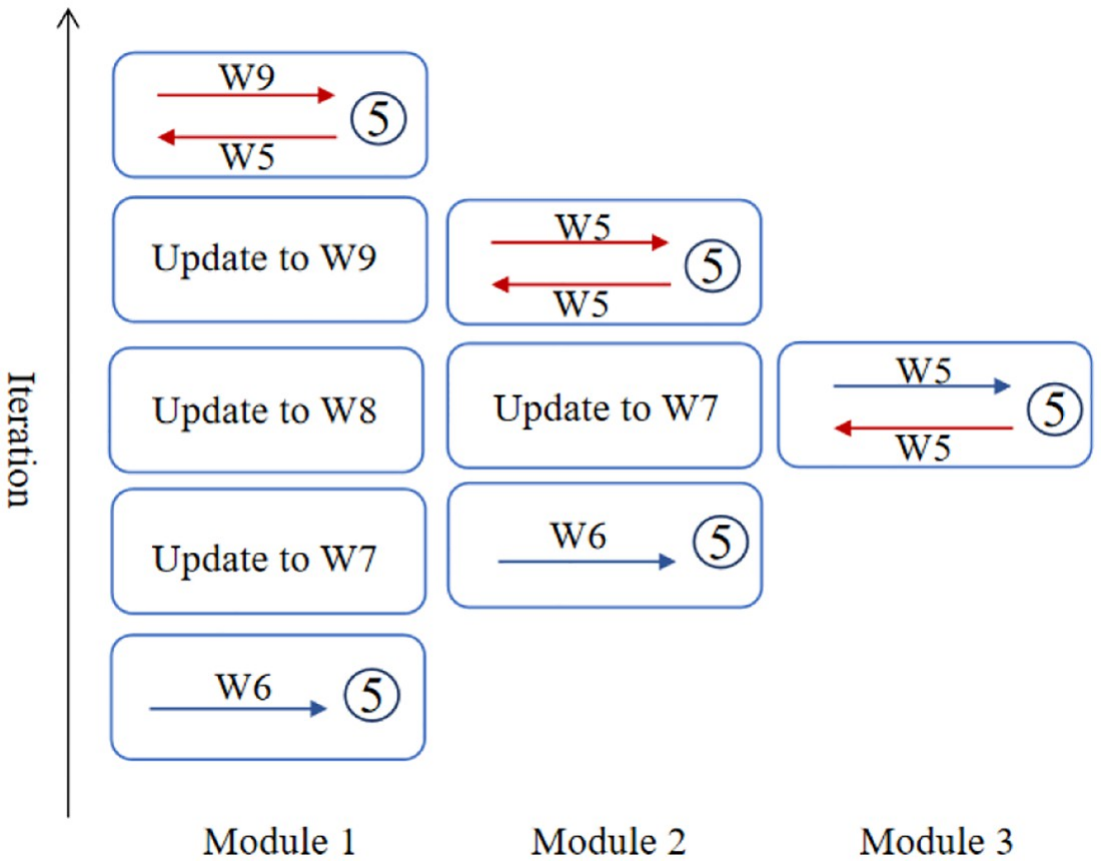
An example of weight delay between two forward passes.

This can also be seen from Fig 1. Alternatively, as shown in Fig 2, taking batch 5 as an example, at *m*_1_, the first FP is computed based on *W*6, while the second BP is executed based on *W*9. A 3-step difference in weights is observed. At *m*_2_, there is a 1-step difference in weights.

To address the weight staleness problem, a weight prediction scheme is proposed to reduce the difference between the weights of first FP and second FP. The weight prediction will be executed during the first FP to predict the weight to bring the values closer to that of the second. This can be shown as follows:

$$\begin{array}{c} m_{k}\left(w^{t-k+2}\to w^{p}\right)\to a_{l_{k,L_{k}}}^{t-k+1}. \end{array}$$ (14)

Therefore, the activations generated during the first FP, in which the predicted weight is used, will be closer to activations produced during the second FP and hence the weight staleness effect can be mitigated. It will be beneficial to alleviate the performance drop caused by delayed weights. Moreover, the process of weight prediction can implicitly add noise during the training so that the trained model could enhance its generalization ability. The proposed decoupled network training with re-computation and weight prediction (DTRP) is summarized in Algorithm 1. The remaining part in this section introduces the weight predictor in detail.

**Algorithm 1:** DTRP

**Input**: A set of training samples split into mini-batches *x*.

**Required**: Learning rate *η*, gradient shrinking factor *β*.

• Split the model into *K* modules: **for** iteration *t* = 1: *T*:

 **for** each module *k* = 1: *K* in parallel:

  **if** *k* ≠ *K*:

   Backward stage:

   • Execute forward pass and output ${\tilde{a}}_{l_{k,L_{k}}}^{t+k-2K+1}$ (except for last module).

   • Execute backward pass and compute delayed gradient for each layer ${\hat{g}}_{w_{l}}^{t-k+1}$ and gradient for previous layer ${\hat{g}}_{a_{l_{k-1,L_{k-1}}}}^{t+k-2K+1}$.

   • Update model weight.

   Forward stage:

   •Weight Prediction: ***w***^*t*−*k*+2^ → ***w***^*p*^.

   •Execute forward pass and output $a_{l_{k,L_{k}}}^{t-k+1}$.

   •Reload previous weight ***w***^*t*−*k*+2^.

  **else**: (*k* = *K*)

   • Execute forward pass and output $a_{l_{K,L_{K}}}^{t-K+1}$.

   • Execute backward pass and weight update and generate ${\hat{g}}_{a_{l_{K-1,L_{K-1}}}}^{t-K+1}$.

  Communication:

   •Pass $a_{l_{k,L_{k}}}^{t-k+1}$ to *m*_*k*_.

   •Pass ${\hat{g}}_{a_{l_{k-1,L_{k-1}}}}^{t+k-2K+1}$ to *m*_*k*−1_.

 **End for**

**End for**

#### 3.2.1 Weight delay regulator

If the weight delay increases, the number of updates that the module goes through will also increase. It is then more difficult to correctly predict the weight from many iterations away. Hence, we should not rely heavily on the weight prediction based on previous observations. Otherwise the training may not converge. Therefore, we need to regulate the delay multiplier (we refer to the weight delay steps as delay multiplier denoted by *delay* in the weight predictor) to prevent it from being too large in the case when the delay is significant. The weight delay regulator can be expressed as

$$\begin{array}{c} f\left(delay\right)=\{\begin{array}{ccc} delay & & delay\leq tp\\ tp+\text{ln}(delay-e) & & delay>tp \end{array} \end{array}$$ (15)

where *e* ≈ 2.71828 is the natural number, *tp* denotes the turning point and ln() is the natural logarithm function. In this paper, *tp* is set to 3.

**Fig 3:**
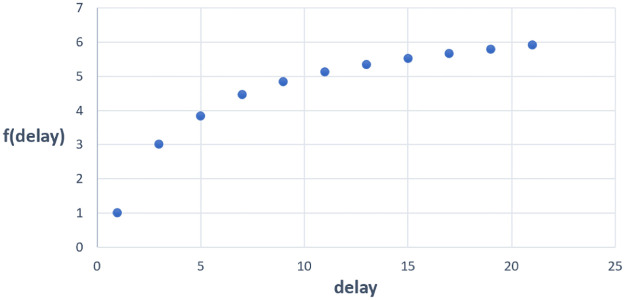
Visualization of delay regulator.

Intuitively, this function is to constrain the delay multiplier in excess of the turning point (visualized in Fig 3). The function is plotted in discrete space because delay can only be odd integers. In the following section, the predictor will use the delay regulator (referred to as *f*(*delay*)) to restrict the delay multiplier.

#### 3.2.2 EMA-Adam predictor

We treat the prediction as a special case of training neural network’s weights. Here we introduce the exponential moving average—Adam (EMA-Adam) predictor that combines the exponential average of gradient and the Adam optimizer [26]. The Adam optimizer has been proven to work well in various optimization applications especially in training neural networks. The gradient at the current iteration is smoothened by taking the exponential average of past gradients before performing steps in Adam optimizer as shown below:

$$\begin{array}{c} {\bar{g}}_{t}=0.6{\bar{g}}_{t-1}+0.4g_{t} \end{array}$$ (16)

where ${\bar{g}}_{t}$ is used to denote the averaged gradient.

Next, ${\bar{g}}_{t}$ will be substituted into the Adam optimizer. Adam optimizer combines the momentum and the RMSprop algorithm [27] which calculates both the exponential average of the past squared gradients and also the exponential average of past gradients. The gradient obtained from Adam optimizer is subsequently multiplied by the regulated delay multiplier to predict the new weight. The predictor can be summarized as follows:

$$\begin{array}{c} V_{t}=\beta_{1}V_{t-1}+\left(1-\beta_{1}\right){\bar{g}}_{t} \end{array}$$ (17)

$$\begin{array}{c} S_{t}=\beta_{2}S_{t-1}+\left(1-\beta_{2}\right){\bar{g}}_{t}^{2} \end{array}$$ (18)

$$\begin{array}{c} V_{t}^{corrected}=V_{t}/\left(1-\beta_{1}^{t}\right) \end{array}$$ (19)

$$\begin{array}{c} S_{t}^{corrected}=S_{t}/\left(1-\beta_{2}^{t}\right) \end{array}$$ (20)

$$\begin{array}{c} \Delta w_{t}=-\eta_{t}\frac{V_{t}^{corrected}}{\sqrt{S_{t}^{corrected}}+\epsilon } \end{array}$$ (21)

$$\begin{array}{c} w_{p}=w_{t}+f\left(delay\right)\Delta w_{t} \end{array}$$ (22)

where $V_{t}^{corrected}$ and $S_{t}^{corrected}$ are the corrected values for *V*_*t*_ and *S*_*t*_ after bias correction to solve the inaccurate estimation caused by the inaccurate initialization of *V*_*t*_ and *S*_*t*_. *β*_1_ and *β*_2_ are set to the default values which are 0.9 and 0.999, respectively. *ϵ* is set to 10^−8^.

The obtained Δ*w*_*t*_ from both averaged squared gradient and averaged gradient will smoothen the gradients across different iterations and is used to indicate the predicted degree of weight update to predict the future weights. In this case, a more lagging double-smoothed gradient is used to indicate the degree of difference between weights at consecutive iterations. In the event of gradient descent to use the gradients to guide the direction towards the local minimum, a greedy step is taken to obtain lower loss. Because of the variance between different mini-batches, the direction may fluctuate frequently. However, in the event of weight prediction, it is desirable to know the general direction that the weight is updated so that the predicted weight does not deviate significantly from the actual weight in the future iterations, thereby avoiding divergence. Hence, employing a smoothened and averaged past degree of update could possibly produce a more promising prediction.

### 3.3 Batch compensation

Ideally, if the network can be divided equally in order to give an equal computation load to each worker, a theoretical maximum acceleration can be achieved. However, in real applications it is hard to evenly split the model. The amount of computation in each layer varies in a network. For instance, in a convolutional neural network, earlier layers may have a larger amount of calculation because they have to process larger feature maps. Since the model is divided according to the basic unit of one layer instead of partitioning at arbitrary positions, it is difficult to ensure an equal distribution of computation load among modules. Hence, the speed of training is mainly determined by the module that requires most computations and time to complete an iteration of learning due to the barrel effect.

**Fig 4:**
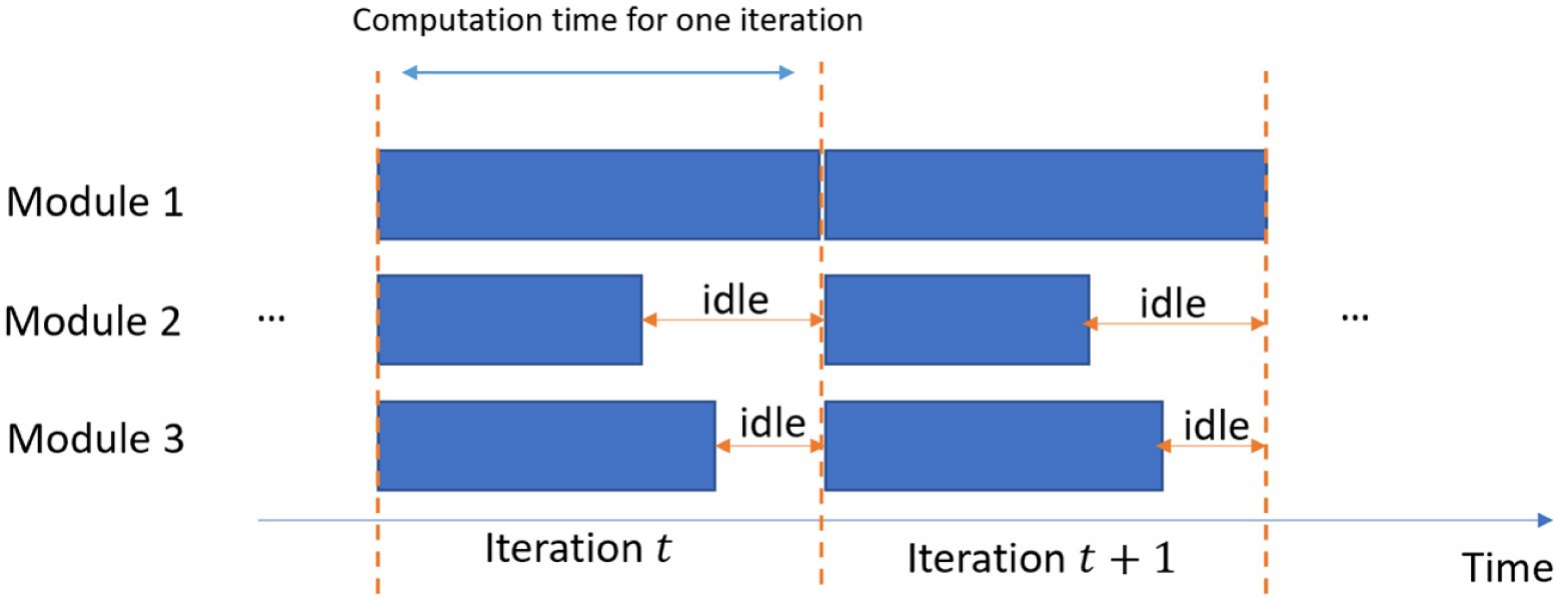
Example of module imbalance.

As shown in Fig 4, three modules have different computation time for one iteration, respectively. After module 2 and module 3 finished one iteration of training, they remain idle and have to wait for module 1 to finish the computation until they can start the next iteration of running. Hence, alleviating the unbalanced distribution of computation is another aspect that can be possibly explored on top of the DTRP to allow more efficient training. Here, a batch compensation method is proposed to mitigate the imbalanced computation load and accelerate the data processing.

The basic idea of batch compensation is to reduce the batch size in specific modules that are distributed with much heavier computation workload. To fully understand the compensation intuition, we need to know how each worker (take GPU as an example) is utilized in the following subsection.

#### 3.3.1 GPU utilization

**Fig 5:**
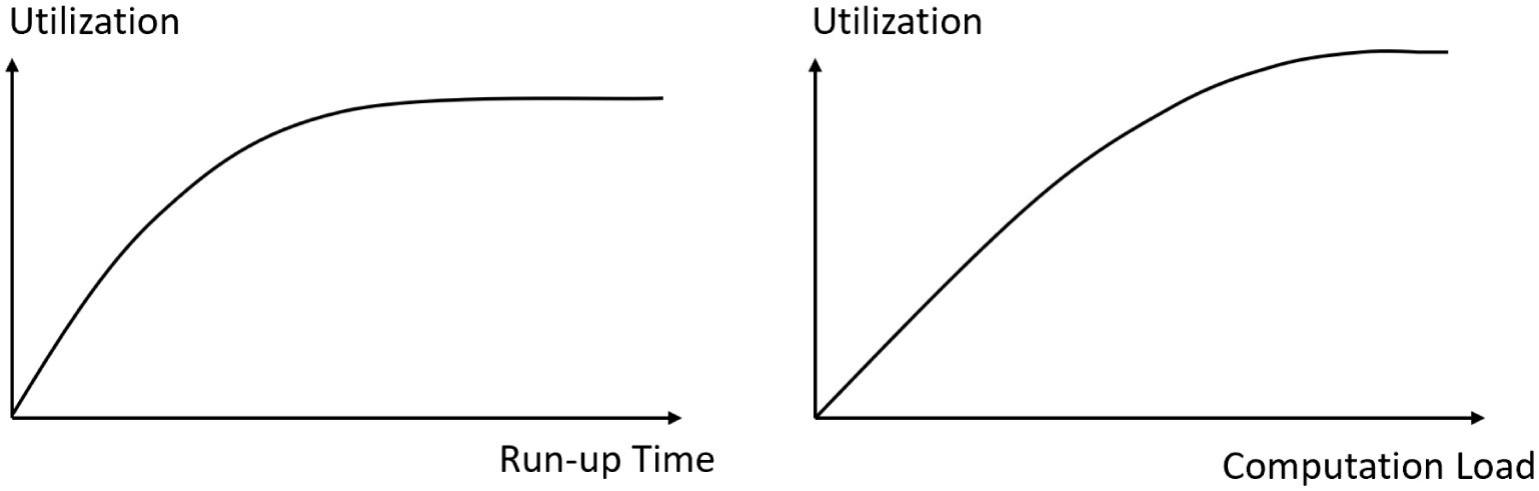
GPU behavior.

Before showing the compensation technique, some basic knowledge of GPU utilization is introduced. The batch compensation method is based on the behavior of GPU utilization [28]. The GPU volatile utilization reports the percentage of time that GPU kernels are active given a time period. When a GPU starts computing, its utilization of behaves like that in Fig 5. The utilization of GPU will gradually increase until saturate (i.e., the steady state). The steady-state utilization level depends on the computation load of the work. If a processor becomes idle for a certain amount of time, it will take time to gradually increase its utilization and processing speed to reach the steady state again. Therefore, to accelerate its processing speed, GPU has to maintain busy at all time, avoiding pauses if possible.

GPU utilization with respect to the computation load behaves in a similar way. When the computation load is light, GPU utilization is sub-optimal, and it does not run at full speed. With the increase of computation load, GPU utilization grows until reaching 100% utilization and the processor will run at full speed. It is worth noting that the slope at the sub-optimal utilization segment in the curve is higher than the segment near optimal utilization. Hence, in the situation of training a neural network, when the batch size at every iteration increases, the utilization of GPU will increase and hence the training process can be accelerated. When the GPU utilization is high, because of the small slope at the optimal utilization segment in the curve, decreasing the batch size slightly will not affect the GPU utilization significantly.

#### 3.3.2 Implementation of batch compensation

**Fig 6:**
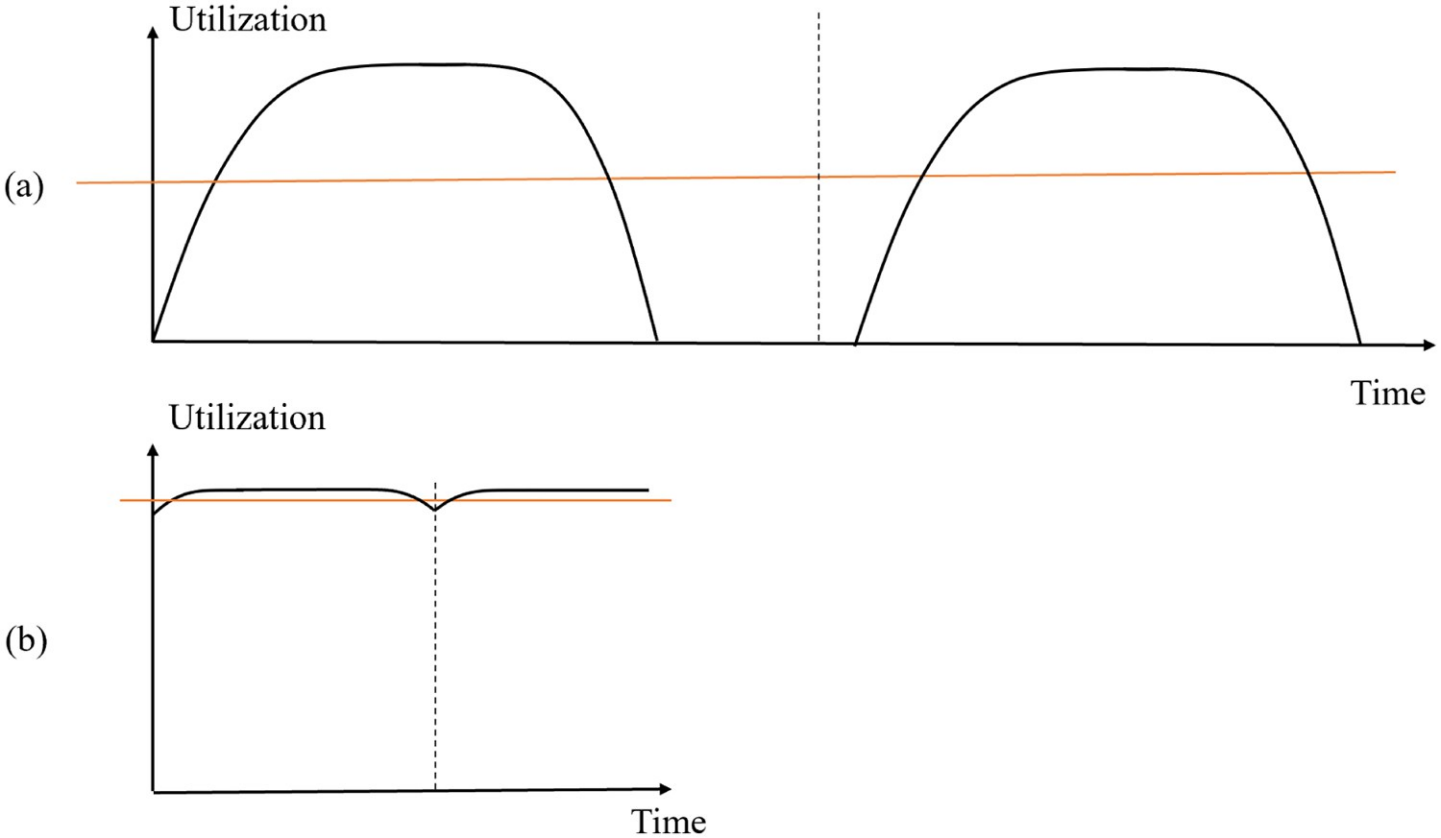
Utilization level without batch compensation (red line: Average utilization level).

At each iteration, all modules have to wait for the one that takes the longest time to finish the iteration. Before other GPUs finish their training iterations, GPUs with lighter computation load stay idle and their utilization may even drop to 0. When the module with the heaviest load finishes the computation, other GPUs have to restart the kernel and gradually increase to the steady state in utilization. The average utilization level will be low as shown in Fig 6(a). This causes decrease in the overall speed.

Hence, batch compensation is proposed by reducing the batch size by a small percentage so that the training time per iteration of each module becomes more balanced. Other modules do not need to wait for the slowest one and pause the GPU for a long time. Since the utilization of these GPUs only decreases by a small percentage, it takes less time for them to ramp up to the steady state as shown in Fig 6(b). In this way, the GPUs are kept busy most of the time. GPUs could run at a higher utilization level on average throughout the training. This could accelerate the training process to a certain extent by increasing the overall speed and makes full use of the computation resources.

Specifically, a drop factor *d* will be used to indicate the percentage of training data in a batch that will be dropped during the training of the slowest module. For every batch of data, during the first FP, all modules will process the same amount of data to produce the final output at the last layer. During the backward stage, {d*batch size} of data will be dropped in the slowest module to reduce computation. The samples being dropped are chosen according to their produced loss at the final layer so that we only drop samples having the lowest loss. Hence, the model can learn more from those difficult samples and skip the backward pass of those well-learned and easy samples.

### 3.4 Comparison with FDG

Although DTRP shares several similarities with FDG [21], DTRP outperforms FDG and solves its limitations. While FDG suffers from the memory exploding problem with the increased number of module splits and the imbalance load of across modules, we summarize the improvement and uniqueness of DTRP as distinguished from FDG:

- We develop a re-computation scheme to free up the memory of each module from storing activations in previous forward stages in each round of backpropagation.
- On top of re-computation method, we propose a weight prediction method to mitigate the stale weight problem introduced by re-computation and boost the performance shown by various experiments.
- A pioneering solution for imbalanced load across modules, named as batch compensation, is also proposed.

Furthermore, we provide a numerical comparison among various decoupled learning methods through experiments in Section 5 and show that our proposed DTRP achieve a state-of-the-art performance on different models and datasets.

## 4 Convergence analysis

In this section, we provide the convergence analysis for the proposed DTRP. We first rewrite all related parameters and gradients into the following form for convenience:

$$\begin{array}{c} w^{t}=\left[{\left(w_{m_{1}}^{t}\right)}^{T},\ldots ,{\left(w_{m_{K}}^{t}\right)}^{T}\right] \end{array}$$ (23)

$$\begin{array}{c} w_{m_{k}}^{t}=\left[{\left(w_{l_{k,1}}^{t}\right)}^{T},\ldots ,{\left(w_{l_{k,L_{k}}}^{t}\right)}^{T}\right] \end{array}$$ (24)

$$\begin{array}{c} g_{w}^{t}=\left[{\left(g_{w_{m_{1}}}^{t}\right)}^{T},\ldots ,{\left(g_{w_{m_{K}}}^{t}\right)}^{T}\right] \end{array}$$ (25)

$$\begin{array}{c} g_{w_{m_{k}}}^{t}=\left[{\left(g_{w_{l_{k,1}}}^{t}\right)}^{T},\ldots ,{\left(g_{w_{l_{k,L_{k}}}}^{t}\right)}^{T}\right] \end{array}$$ (26)

$$\begin{array}{c} {\bar{g}}_{w}^{t}=\left[{\left({\bar{g}}_{w_{m_{1}}}^{t}\right)}^{T},\ldots ,{\left({\bar{g}}_{w_{m_{K}}}^{t}\right)}^{T}\right] \end{array}$$ (27)

$$\begin{array}{c} {\bar{g}}_{w_{m_{k}}}^{t}=\left[{\left({\bar{g}}_{w_{l_{k,1}}}^{t}\right)}^{T},\ldots ,{\left({\bar{g}}_{w_{l_{k,L_{k}}}}^{t}\right)}^{T}\right] \end{array}$$ (28)

where ${\bar{g}}_{w}^{t}$ denotes the full gradient which is obtained by

$$\begin{array}{c} {\bar{g}}_{w}^{t}=\frac{\partial L(w^{t})}{\partial w^{t}}. \end{array}$$ (29)

To simplify the following convergence analysis, we replace *t* − *k* + 1 with *t* in the formula (12) since for each module *m*_*k*_ at iteration *t* + *k* − 1, the corresponding activation index is *t*. Thus, the new weight updating formula is shown as

$$\begin{array}{c} w_{l}^{t+1}=w_{l}^{t}-\gamma \eta_{t}{\hat{g}}_{w_{l}}^{t}. \end{array}$$ (30)

The gradient calculation formula (9) is also shifted to

$$\begin{array}{c} {\hat{g}}_{w_{l}}^{t}={\hat{g}}_{a_{l_{k,L_{k}}}}^{t+2(k-K)}\frac{\partial {\tilde{a}}_{l_{k,L_{k}}}^{t+2(k-K)}}{\partial w_{l}^{t}}. \end{array}$$ (31)

By defining *d*_*k*,*t*_ = *t* + 2(*k* − *K*), the gradient calculation formula can further be written as

$$\begin{array}{c} {\hat{g}}_{w_{l}}^{t}={\hat{g}}_{a_{l_{k,L_{k}}}}^{d_{k,t}}\frac{\partial {\tilde{a}}_{l_{k,L_{k}}}^{d_{k,t}}}{\partial w_{l}^{t}}. \end{array}$$ (32)

Note that for the following assumptions, the subscript *l* of weights ***w***_*l*_ denotes the gradients is layer-wise and all conclusions can be generalized to both module-wise $w_{m_{k}}$ and model-wise ***w***.

*Assumption 1*: Following the Assumption 1 (*Sufficient direction*) made in [18], we assume that the descent direction ${\hat{g}}_{w_{l}}^{t}$ which is obtained after the second forward in the DTRP algorithm, i.e., based on the updated weights $w_{l}^{t}$ is a *sufficient descent direction* w.r.t to the delayed SGD gradient $g_{w_{l}}^{d_{k,t}}$:

$$\begin{array}{c} \begin{array}{c} {\hat{g}}_{w_{l}}^{t}={\hat{g}}_{a_{l_{k,L_{k}}}}^{d_{k,t}}\frac{\partial {\tilde{a}}_{l_{k,L_{k}}}^{d_{k,t}}}{\partial w_{l}^{t}}=\frac{\partial {\tilde{a}}_{l_{k,L_{k}}}^{d_{k,t}}}{\partial w_{l}^{t}}\frac{\partial f_{x^{d_{k,t}}}(w^{d_{k,t}})}{\partial {\tilde{a}}_{l_{k},L_{k}}^{d_{k,t}}}\\ =\frac{\partial a_{l_{k,L_{k}}}^{d_{k,t}}}{\partial w_{l}^{d_{k,t}}}\frac{\partial f_{x^{d_{k,t}}}(w^{d_{k,t}})}{\partial a_{l_{k},L_{k}}^{d_{k,t}}}+\Delta g_{w_{l}}^{t}=g_{w_{l}}^{d_{k,t}}+\Delta g_{w_{l}}^{t} \end{array} \end{array}$$ (33)

where we treat $\Delta g_{w_{l}}^{t}$ as a zero-mean disturbance, i.e., $E[\Delta g_{w_{l}}^{t}]=0$ and there exists a constant *M*_*g*_ ≥ 0 so that $E[||\Delta g_{w_{l}}^{t}{\left|\right|}_{2}^{2}]\leq M_{g}$.

The following two assumptions are commonly used in neural network convergence analysis according to the studies in [17, 29].

*Assumption 2*: We assume the gradient of the loss function throughout the paper is *Lipschitz continuous gradient*, which means that for two gradients at different time stamps, there exists a constant *L* > 0 such that

$$\begin{array}{c} \left|\right|{\bar{g}}_{w}^{t_{1}}-{\bar{g}}_{w}^{t_{2}}{\left|\right|}_{2}\leq L||w^{t_{1}}-w^{t_{2}}{\left|\right|}_{2} \end{array}$$ (34)

$$\begin{array}{c} \left|\right|g_{w_{m_{k}}}^{t_{1}}-g_{w_{m_{k}}}^{t_{2}}{\left|\right|}_{2}\leq L||w_{m_{k}}^{t_{1}}-w_{m_{k}}^{t_{2}}{\left|\right|}_{2}. \end{array}$$ (35)

*Assumption 3*: We further assume that the variance of SGD gradients are upper bounded. Thus for ∀*t*, ∃*M* > 0 such that:

$$\begin{array}{c} \left|\right|g_{w}^{t}\left|\right|\leq M. \end{array}$$ (36)

Holding these assumptions, we obtain three theorems which prove our proposed DTRP algorithm can converge to the critical point.

*Theorem 1*: Let Assumption 1 to 3 hold true. Assuming further that the shrink learning rate *γη*_*t*_ is designed that *Lγη*_*t*_ ≤ 1, we can derive the following inequality:

$$\begin{array}{c} \begin{array}{cc} E_{x_{t}}[f(w^{t+1})]-f(w^{t})\leq & -\gamma \eta_{t}\sum_{k=1}^{K}{∥{\bar{g}}_{w_{m_{k}}}^{t}∥}_{2}^{2}\\ & +{\left(\gamma \eta_{t}\right)}^{2}(L\left(2M+M_{g}\right)K+LM\sum_{k=1}^{K}3\left(K-k\right)). \end{array} \end{array}$$ (37)

From the Eq (37), we conclude that the expectation of loss will decrease if the right side is negative:

$$\begin{array}{c} -\gamma \eta_{t}\sum_{k=1}^{K}{∥{\bar{g}}_{w_{m_{k}}}^{t}∥}_{2}^{2}+{\left(\gamma \eta_{t}\right)}^{2}(L\left(2M+M_{g}\right)K+LM\sum_{k=1}^{K}3\left(K-k\right))<0 \end{array}$$ (38)

$$\begin{array}{c} \gamma \eta_{t}<min\{\frac{1}{L},\frac{\sum_{k=1}^{K}{∥{\bar{g}}_{w_{m_{k}}}^{t}∥}_{2}^{2}}{\left(L\left(2M+M_{g}\right)K+LM\sum_{k=1}^{K}3\left(K-k\right)\right)}\}. \end{array}$$ (39)

*Theorem 2*: Holding Assumption 1 to 3, denote w* as the critical point of the loss function and $T_{T}=\sum_{t=0}^{T-1}\gamma \eta_{t}$. Based on Theorem 1, we can further derive that

$$\begin{array}{c} \begin{array}{cc} \frac{1}{T_{T}}\sum_{t=0}^{T-1}\gamma \eta_{t}E{∥{\bar{g}}_{w}^{t}∥}_{2}^{2}\leq & \frac{f(w^{0})-f(w^{*})}{T_{T}}\\ & +\frac{(L\left(2M+M_{g}\right)K+LM\sum_{k=1}^{K}3\left(K-k\right))\sum_{t=0}^{T-1}\gamma \eta_{t}^{2}}{T_{T}}. \end{array} \end{array}$$ (40)

Theorem 2 indicates that if the nonincreasing learning rate satisfies that $\lim_{T\to \infty }T_{T}=\infty$ and $\lim_{T\to \infty }{\left(\gamma \eta_{t}\right)}^{2}<\infty$, randomly sampling *w*^*z*^ where *z* = 0, 1, …, *T* − 1 with the probability $\frac{\gamma \eta_{z}}{T_{T}}$, its gradient satisfy

$$\begin{array}{c} \lim_{T\to \infty }E{∥{\bar{g}}_{w}^{z}∥}_{2}^{2}=0 \end{array}$$ (41)

which proves that the algorithm converges to the critical point.

*Theorem 3*: Assume that Assumption 1 to 3 hold and set the learning rate to be a constant $\gamma \eta_{t}=\gamma \eta =\sqrt{\frac{{∊}^{2}(f(w^{0})-f(w^{*}))}{T(L\left(2M+M_{g}\right)K+LM\sum_{k=1}^{K}3\left(K-k\right))}}$ which leads Theorem 2 to a special case, we have

$$\begin{array}{c} \begin{array}{cc} & \underset{t\in \{0,1,\ldots ,T-1\}}{\text{min}}E[{∥{\bar{g}}_{w}^{t}∥}_{2}^{2}]\\ & \leq \frac{\left(1+\epsilon^{2}\right)}{\epsilon }\sqrt{\frac{\left(f(w^{0})-f(w^{*}))(L\left(2M+M_{g}\right)K+LM\sum_{k=1}^{K}3\left(K-k\right)\right)}{T}}. \end{array} \end{array}$$ (42)

Theorem 3 shows that DTRP converges to the critical point with a well-designed constant learning rate.

The detailed proofs of the above theorems are provided in the S1 File.

## 5 Experiments and results

In this section, experiments are conducted on benchmark datasets including CIFAR10, CIFAR100 [30] and ImageNet [31]. Different CNNs are trained to perform image classification tasks to evaluate the proposed DTRP method. The results of the proposed DTRP are reported in terms of accuracy, memory usage and acceleration. These experiments show that the DTRP produces comparable results with standard BP while solving the exploding memory problem and achieves significant acceleration. Our experiments begin at validating the DTRP’s alleviation of memory issue, then move to the generalization performance including the weight predictor empirical analysis, and finally end with the evaluation of training speed.

• **Implementation details**: The DTRP algorithm is implemented using PyTorch Library. The network is split into *K* modules and placed on different GPUs. The same training strategy as in FDG [21] is followed. The training images are pre-processed by normalization and standard data augmentation strategies such as random cropping and random horizontal flipping [7, 8]. SGD optimizer [23] is deployed with a learning rate of 0.1 and batch size of 128. The momentum and weight decay are adopted and are chosen to be 0.9 and 0.0005, respectively. The learning rate is decayed by 10 times at epoch number 150, 225, 275. The models are trained for 300 epochs. The generalization performance is reported at the final epoch so no validation set is used in this experiment. For the ImageNet dataset, the models are trained for 90 epochs with learning rate decayed at epoch number 30, 60 and 80. A learning rate of 0.01 is used to warm up [32] the training for 3 epochs. The weight decay is set to 0.0001. *Kaiming Initialization* is adopted to initialize the network [33].

### 5.1 Memory usage analysis

**Fig 7:**
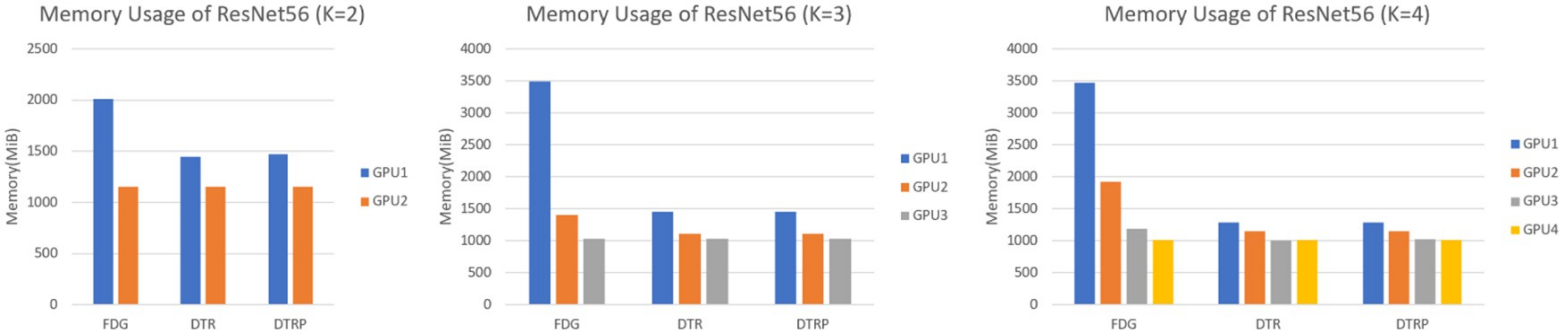
Memory usage of ResNet56 with different *K*s.

To assess the memory performance, the memory usages to train ResNet56 when *K* = 2, 3, 4 in each GPU using different training methods are plotted in Fig 7. The memory usages are recorded in MiB with different colors of bars indicating memory usages in different GPUs. Split module 1, 2, 3, 4 are placed on GPU 1, 2, 3, 4, respectively.

It is observed that with the increase in split number *K*, the memory explosion problem in FDG [21] becomes more evident. Using re-computation, the memory usages in DTRP only differ gently across different GPUs. In particular, the deployment of the weight prediction scheme does not introduce apparent memory occupation. This indicates that the exploding memory problem can be solved effectively through re-computation. Hence, in real applications, *K* can be increased without concerning the memory constraints of the hardware.

### 5.2 Results of weight predictor

**Fig 8:**
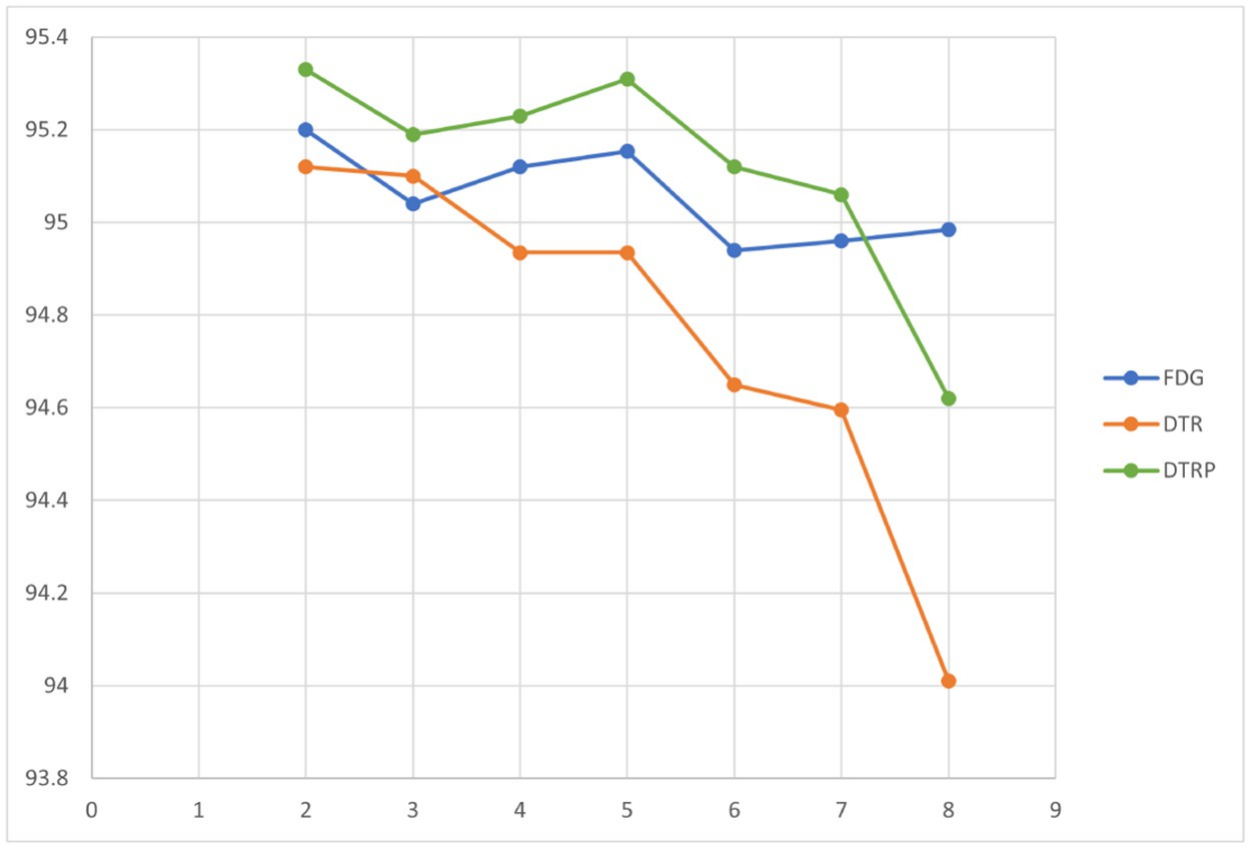
Top-1 accuracy of ResNet18 using different methods.

The Top-1 Accuracy of ResNet18 with with *K* = 1, 2, …, 8 using FDG [21], DTR and DTRP are tabulated in Table 3 with a plot shown in Fig 8. The results are taken from the median of the Top-1 accuracy at the last epoch over 3 times of experiments. dels. FDG [21] also deploys a similar process (named gradient shrinking) to LRS with factor *β* to scale the gradient. The gradient shrinking factor *β* and learning rate shrinking factor *γ* are set to 1 in this experiment. It is observed that the proposed method (i.e., DTR) has marginally lower accuracy compared to FDG. This is due to the effect of delayed weights on the difference in the weights of the forward stage and backward stage during the training of each batch of data.

**Table 3:** Top-1 accuracy (%) of ResNet18 using different methods.

*K*	FDG	DTR	DTRP
**2**	95.20	95.12	**95.33**
**3**	95.04	95.10	**95.19**
**4**	95.12	94.94	**95.23**
**5**	95.15	94.94	**95.31**
**6**	94.94	94.65	**95.12**
**7**	94.96	94.60	**95.06**
**8**	**94.99**	94.01	94.62

However, it can also be observed that with the introduction of the weight prediction scheme in DTRP, the accuracy is improved. With the increase of *K*, the weight delay increases, and the performance loss caused by the stale weight is more visible. The deployment of weight prediction tends to allow more evident improvements when *K* is large. This proves that the weight prediction could enhance the generalization ability of the models and it plays an essential role to mitigate the staleness of weights. Furthermore, when *K* < 8, DTRP even outperforms the state-of-the-art method FDG. This implies that weight prediction can implicitly introduce noise with unknown distributions during training and hence improve the generalization ability of the models. Since FDG applies relatively correct gradients to optimize the models, the increase of *K* does not affect the accuracy significantly while DTRP uses predicted weight that tends to deteriorate as *K* increases. However, FDG suffers from the memory explosion problem at large *K* which is hard to deploy in real applications. DTRP provides a solution to resolve it while maintaining comparable or better generalization results for small and medium *K*.

Table 4 shows the Top-1 accuracy of models including ResNet20 and ResNet56 on datasets including CIFAR10 and CIFAR100. It is observed that DTRP still outperforms DTR for other models on various datasets. This further validates that the adoption of the weight prediction scheme can improve the performance of DTR by mitigating the delay in weight.

**Table 4:** Top-1 accuracy (%) for models with different *K* on CIFAR10 and CIFAR100.

Model	Dataset	DTR	DTRP
[0.3mm] ResNet20(*K* = 2)	CIFAR10	92.58	**92.77**
ResNet56(*K* = 2)	CIFAR100	72.32	**73.02**
ResNet56(*K* = 3)	CIFAR100	72.57	**73.27**
ResNet56(*K* = 4)	CIFAR100	72.57	**73.11**

### 5.3 Comparison of performance on various datasets and models

The generalization performance of DTRP on various datasets and models are reported in this section in comparison with other state-of-the-art methods (including the DDG [17], FR [18], DGL [20], and FDG [21]) and the standard BP. To have fair comparisons, the median of the Top-1 errors at the last epoch over 3 times of experiments is taken, the same as that in [21].

- **CIFAR10**: Table 5(a) shows the Top-1 errors among various models on CIFAR10 when *K* = 2. It is observed that without the LRS process (*γ* = 1), for ResNet110, ResNet18, ResNet1202 and WRN-28–10, DTRP outperforms other methods including BP. For ResNet20 and ResNet18 the proposed DTRP gives better generalization ability than the BP baselines and produces comparable results against other methods. The adoption of LRS process increases the robustness of DTRP. Through setting suitable shrinking factor *γ*, the DTRP outperforms all other methods among all the models. It is also worth noting that when the shrinking factor of both FDG (*β*) and DTRP (*γ*) are set to 1, the DTRP produces better results than FDG for all the models. When the gradient shrinking process and the LRS process are applied respectively, DTRP still outperforms FDG with gradient shrinking process for all the models. It is also shown that DTRP can be applied to CNN models with various depths and widths. It can be trained on rather shallow networks such as ResNet20, the wider network such as WRN-28–10 and extremely deep networks such as ResNet1202.
- **CIFAR100**: The classification performances of different methods are also studied using the CIFAR100 dataset. Table 5(b) tabulated the Top-1 errors of various models on CIFAR100 when *K* = 2. Without the LRS process, for ResNet18 and WRN-28–10, the proposed DTRP produces the best results among all the comapred methods. For the other models, DTRP still produce comparable results. However, with the introduction of LRS process, DTRP again outperforms all other methods for all the models.
- **ImageNet**: The DTRP are also evaluated on ImageNet with 1.33 million images and 1000 classes to be classified. Top-1 and Top-5 errors of ResNet18 and ResNet50 are tabulated in Table 5(c). Similarly, the DTRP outperforms the compared methods in terms of both Top-1 and Top-5 errors. It also denotes that the DTRP can be applied to large-scale datasets.
- **Larger split number**: In this experiment, the generalization performances of WRN-28–10 and ResNet56 with different split numbers *K* are studied. Table 6 shows the Top-1 errors on CIFAR10 with split number *K* equal to 2, 3 or 4. It is observed that for all methods, the increase of *K* causes performance loss. However, the proposed DTRP still outperforms other methods including the BP for all the above-tabulated models when *K* < 3. When *K* is increased to 4, the deployment of learning rate shrinking process mitigate the affect of large *K* and restore the result that are still comparable with BP baseline in ResNet-56.

**Table 5:** The validation errors (%) of the compared methods on (a) CIFAR10, (b) CIFAR100, and (c) ImageNet for *K* = 2. Results with * are rerun using our training strategy, while those without * are the results reported in their original papers [17, 18, 20]. *β* represents the gradient shrinking factor used in FDG [21] and *γ* denotes the learning rate shrinking factors in DTRP.

**Dataset**	**Model**	**# params**	**BP** [14]	**DDG** [17]	**DGL** [20]	**FR** [18]	**FDG**	**DTRP**
**(a) CIFAR10**	ResNet-20	0.27M	8.75%/7.78%*	-	-	-	7.92%*(*β*=1)/7.23%*(*β*=0.2)	7.5%(*γ*=1)/**7.11**%(*γ*=0.5)
	ResNet-56	0.46M	6.97%/6.19%*	6.89%/6.63%*	6.77%*	6.07%*	6.20%*(*β*=1)/5.90%*(*β*=0.5)	5.99%(*γ*=1)/**5.75**%(*γ*=0.8)
	ResNet-110	1.70M	6.43%/5.79%*	6.59%/6.26%*	6.50%/6.26%*	5.76%*	5.79%*(*β*=1)/5.73%*(*β*=0.2)	5.66%(*γ*=1)/**5.17**%(*γ*=0.7)
	ResNet-18	11.2M	6.48%/4.87%*	5.00%*	5.21%*	4.80%*	4.82%*(*β*=1)/4.79%*(*β*=0.8)	4.79%(*γ*=1)/**4.46**%(*γ*=0.4)
	ResNet-1202	19.4M	7.93%/5.51%*	-	-	-	5.50%*(*β*=1)/5.49%*(*β*=0.5)	5.48%(*γ*=1)/**5.12**%(*γ*=0.5)
	WRN-28–10	36.5M	4.00%/4.01%*	4.05%	4.12%	3.87%	4.13%*(*β*=1)/3.85%*(*β*=0.7)	3.68%(*γ*=1)/**3.64**%(*γ*=0.6)
**Dataset**	**Model**	**# params**	**BP [14]**	**DDG** [17]	**DGL** [20]	**FR** [18]	**FDG**	**DTRP**
**(b) CIFAR100**	ResNet-56	0.46M	30.21%/27.68%*	29.83%/28.44%*	29.51%*	28.39%*	27.87%*(*β*=1)/27.49%*(*β*=0.4)	27.88%(*γ*=1)/**27.14**%(*γ*=0.6)
	ResNet-110	1.70M	28.10%/25.82%*	28.61%/27.16%*	26.80%*	26.31%*	25.73%*(*β*=1)/25.43%*(*β*=0.5)	26.03%(*γ*=1)/**25.28**%(*γ*=0.5)
	ResNet-18	11.2M	22.35%*	22.74%*	22.24%*	22.88%*	22.78%*(*β*=1)/22.18%*(*β*=0.5)	21.90%(*γ*=1)/**20.87**%(*γ*=0.4)
	WRN-28–10	36.5M	19.2%/19.6%*	-	-	-	20.28%*(*β*=1)/19.08%*(*β*=0.6)	19.20%(*γ*=1)/**18.09**%(*γ*=0.4)
**Dataset**	**Model**	**# param**	**BP** [14]		**FDG**		**DTRP**	
			Top-1	Top-5	Top-1	Top-5	Top-1	Top-5
**(c) ImageNet**	ResNet-18	11.2M	29.79%*	10.92%*	29.72%*(*β*=1)/29.60%*(*β*=0.3)	10.42%*(*β*=1)/10.51%*(*β*=0.3)	29.66%(*γ*=1)/**29.52%**(*γ*=0.5)	10.44%(*γ*=1)/**10.30%**(*γ*=0.5)
	ResNet-50	25.6M	23.65%*	7.13%*	23.65%*(*β*=1)/24.08%*(*β*=0.5)	7.23%*(*β*=1)/7.23%*(*β*=0.5)	**23.53%**(*γ*=1)/23.58%(*γ*=0.8)	**6.84%**(*γ*=1)/6.88%(*γ*=0.8)

**Table 6:** Top-1 errors (%) for models with different *K* on CIFAR10.

Model	BP [14]	DDG [17]	DGL [20]	FR [18]	FDG	DTRP
ResNet-56(*K* = 2)	6.19%	6.60%*	6.77%*	6.07%*	6.20%*/5.90%*(*β*=0.5)	5.99%/**5.75**%(*γ*=0.8)
ResNet-56(*K* = 3)	6.19%	6.50%*	8.88%*	6.33%*	6.40%*/6.08%*(*β*=0.2)	5.93%/**5.52%**(*γ*=0.8)
ResNet-56(*K* = 4)	6.19%	6.61%*	9.65%*	6.48%*	6.83%*/6.14%*(*β*=0.3)	6.35%/**5.60%**(*γ*=0.7)
WRN-28–10(*K* = 2)	4.01%	4.05%*	4.12%*	3.87%*	4.13%*/3.85%*(*β*=0.7)	3.68%/**3.64%**(*γ*=0.6)
WRN-28–10(*K* = 3)	4.01%	4.12%*	4.91%*	6.16%*	4.19%*/4.07%*(*β*=0.5)	3.94%/**3.93%**(*γ*=0.9)
WRN-28–10(*K* = 4)	**4.01**%	6.61%*	5.64%*	5.39%*	5.61%*/4.42%*(*β*=0.5)	5.03%/4.30%(*γ*=0.6)

### 5.4 Speed analysis

To analyze the speed acceleration, experiments of training ResNet101 on CIFAR10 are conducted using RTX2080Ti GPUs. The speed of BP, DTRP and DTRP with proposed batch compensation when *K* = 2, 3 are tabulated in Table 7. The DTRP with batch compensation method adopt a drop factor *d* = 0.2. The speeds are reported using unit #images/s and they are measured over a period of 20s during training. For instances, 760.55 (96%, 79%) 1.69× means 760.55 images/s with 2 GPUs at utilizations 96% and 79% respectively, and a 1.69× speed up compared to BP. The experiments are conducted without high-speed interconnect among GPUs.

**Table 7:** Comparison of speed using different methods.

Methods	*K* = 2	*K* = 3
BP	448 (91%)	448 (91%)
DTRP	760.55 (96%, 79%) 1.69×	888.32 (94%, 65%, 63%) 1.98×
DTRP with Batch Compensation	827.64 (94%, 94%) 1.85×	970.69 (93%, 80%, 79%) 2.17×

Several points can be concluded from the table.

1) Compared with BP, DTRP shows significant acceleration in the training process. Since much longer computation time is taken by the backward pass compared to the time taken by the forward pass, the replay of forward pass does not cause the acceleration to mitigate significantly. The DTRP can achieve more than 1.5× speed up when *K* = 2.
2) Compared with DTRP itself, DTRP with batch compensation further accelerates the training process. It is observed that in the DTRP method, the GPU utilizations are more imbalanced across GPUs (see the GPU utilizations in Table 7). This is because of the unequal assignment of computation load to each module. By dropping a small portion of samples, the utilizations are more balanced. The utilizations of those GPUs whose original utilization are high still remain high and those whose original utilizations are rather low have been improved. Hence, the overall utilization is increased and the GPUs can process images at a higher speed in general. This implies that by compensating the imbalanced computation load over different modules by adjusting the batch size, the training process can be executed faster.
3) It is observed that the GPU utilization when running BP does not reach the highest point. This is due to the memory constraint of the GPUs available for this research. However, if DTRP is applied to perform the training, the model can be split into modules and placed on multiple GPUs, giving more memory space to load more data, hence achieving higher GPU utilization and higher speed. This implies that the proposed method can be applied in real applications to solve the problem of limited memory space when training large networks.

Table 8 shows the Top-1 errors of ResNet20 on CIFAR10 with and without the adoption of batch compensation. The drop factor *d* is set to 0.2. It is observed that the Top-1 error of DTRP with batch compensation increases marginally compared to DTRP when *K* = 2. This is inevitable as some samples are removed during the backward stage [34]. This indicates that the adoption of batch compensation can accelerate the training significantly while causing only marginal performance loss.

**Table 8:** Top-1 errors of ResNet20.

Model	DTRP	DTRP with batch compensation
ResNet20 (*K* = 2)	7.3%	7.35%
ResNet20 (*K* = 3)	7.64%	7.38%

Batch compensation provides an alternative to make a trade-off between accuracy and speed. For users who have high requirement on runtime, it offers an option to further accelerate training at the cost of minor accuracy drop. This can be possibly applied during the prototyping stage of development, when rapid re-iteration of model training is necessary. To the author’s best knowledge, this is the first study that attempts to explore potential solutions to mitigate the problem of imbalance split of a model. Therefore, the contribution of this paper could provide a possible direction for subsequent studies on this topic.

## 6 Conclusion

To address the problem of lockings in BP for neural network training, a novel decoupled training with re-computation and weight prediction (DTRP) method has been proposed. The new method divides a model into several modules and trains them asynchronously, hence facilitating model parallelization to accelerate the training of neural networks. The re-computation scheme in the DTRP effectively solves the memory explosion problem through re-executing forward pass. To mitigate the issue of weight staleness, the proposed EMA-Adam weight predictor predicts the weights at the future stages to narrow the delay in weights between two forward passes. A batch compensation scheme is also explored to alleviate the suboptimal utilization of processors caused by imbalanced computation load among split modules, hence achieving further acceleration. Theoretical analysis shows that the DTRP guarantees convergence. Demonstrated by experiments on image classification tasks using CNNs, the DTRP provides comparable or better results than the state-of-the-art methods and even outperforms the standard BP. To conclude, the DTRP effectively solves the exploding memory problem in decoupled learning and achieves significant acceleration.

## Supporting information

S1 File (PDF)
