# Supplementary material for: Decoupled neural network training with re-computation and weight prediction
Source: PLoS One. 2023 Feb 23;18(2):e0276427. doi: 10.1371/journal.pone.0276427 (PMC9949630; doi:10.1371/journal.pone.0276427)
Supplement: S1 File — (PDF) [file pone.0276427.s001.pdf]

Supporting information

S1 Appendix

Proof of Theorem 1. According to Assumption 2 of *Lipschitz Continuous Gradient*, the following inequality holds:

$$f\left(\boldsymbol{w}^{t+1}\right) \leq f\left(\boldsymbol{w}^{t}\right) + \left(\overline{\boldsymbol{g}}_{\boldsymbol{w}}^{t}\right)^{T}\left(\boldsymbol{w}^{t+1} - \boldsymbol{w}^{t}\right) + \frac{L}{2}\left\|\boldsymbol{w}^{t+1} - \boldsymbol{w}^{t}\right\|_{2}^{2}. \tag{43}$$

According to the updating rule (30), Eq. (43) can be rewritten as

$$f\left(\boldsymbol{w}^{t+1}\right) \leq f\left(\boldsymbol{w}^{t}\right) - \gamma\eta_t \sum_{k=1}^{K}\left(\overline{\boldsymbol{g}}_{\boldsymbol{w}_{m_k}}^{t}\right)^{T}\hat{\boldsymbol{g}}_{\boldsymbol{w}_{m_k}}^{t} + \frac{L(\gamma\eta_t)^2}{2}\sum_{k=1}^{K}\left\|\hat{\boldsymbol{g}}_{\boldsymbol{w}_{m_k}}^{t}\right\|_{2}^{2}. \tag{44}$$

Eq. (44) rewrites the dot product of model-wise gradients in (43) into the summation of K module-wise dot products. We further expand $\hat{g}_{\boldsymbol{w}_{m_k}}^{t}$ under Assumption 1:

$$\begin{aligned}
f\left(\boldsymbol{w}^{t+1}\right) &\leq f\left(\boldsymbol{w}^{t}\right) - \gamma\eta_t \sum_{k=1}^{K}\left(\overline{\boldsymbol{g}}_{\boldsymbol{w}_{m_k}}^{t}\right)^{T}\hat{\boldsymbol{g}}_{\boldsymbol{w}_{m_k}}^{t} + \frac{L(\gamma\eta_t)^2}{2}\sum_{k=1}^{K}\left\|\hat{\boldsymbol{g}}_{\boldsymbol{w}_{m_k}}^{t}\right\|_{2}^{2} \\
&= f\left(\boldsymbol{w}^{t}\right) - \gamma\eta_t \sum_{k=1}^{K}\left(\overline{\boldsymbol{g}}_{\boldsymbol{w}_{m_k}}^{t}\right)^{T}\left(\boldsymbol{g}_{\boldsymbol{w}_{m_k}}^{d_k,t} + \Delta g_{\boldsymbol{w}_{m_k}}^{t}\right) + \frac{L(\gamma\eta_t)^2}{2}\sum_{k=1}^{K}\left\|\boldsymbol{g}_{\boldsymbol{w}_{m_k}}^{d_k,t} + \Delta g_{\boldsymbol{w}_{m_k}}^{t}\right\|_{2}^{2} \\
&\leq f\left(\boldsymbol{w}^{t}\right) - \gamma\eta_t \sum_{k=1}^{K}\left(\overline{\boldsymbol{g}}_{\boldsymbol{w}_{m_k}}^{t}\right)^{T}\boldsymbol{g}_{\boldsymbol{w}_{m_k}}^{d_k,t} + L(\gamma\eta_t)^2\sum_{k=1}^{K}\left\|\boldsymbol{g}_{\boldsymbol{w}_{m_k}}^{d_k,t}\right\|_{2}^{2} \\
&- \gamma\eta_t \sum_{k=1}^{K}\left(\overline{\boldsymbol{g}}_{\boldsymbol{w}_{m_k}}^{t}\right)^{T}\Delta g_{\boldsymbol{w}_{m_k}}^{t} + L(\gamma\eta_t)^2\sum_{k=1}^{K}\left\|\Delta g_{\boldsymbol{w}_{m_k}}^{t}\right\|_{2}^{2} \\
&= f\left(\boldsymbol{w}^{t}\right) - \gamma\eta_t \sum_{k=1}^{K}\left(\overline{\boldsymbol{g}}_{\boldsymbol{w}_{m_k}}^{t}\right)^{T}\left(\boldsymbol{g}_{\boldsymbol{w}_{m_k}}^{d_k,t} - \overline{\boldsymbol{g}}_{\boldsymbol{w}_{m_k}}^{t} + \overline{\boldsymbol{g}}_{\boldsymbol{w}_{m_k}}^{t}\right) \\
&+ L(\gamma\eta_t)^2\sum_{k=1}^{K}\left\|\boldsymbol{g}_{\boldsymbol{w}_{m_k}}^{d_k,t} - \overline{\boldsymbol{g}}_{\boldsymbol{w}_{m_k}}^{t} + \overline{\boldsymbol{g}}_{\boldsymbol{w}_{m_k}}^{t}\right\|_{2}^{2} - \gamma\eta_t \sum_{k=1}^{K}\left(\overline{\boldsymbol{g}}_{\boldsymbol{w}_{m_k}}^{t}\right)^{T}\Delta g_{\boldsymbol{w}_{m_k}}^{t} \\
&+ L(\gamma\eta_t)^2\sum_{k=1}^{K}\left\|\Delta g_{\boldsymbol{w}_{m_k}}^{t}\right\|_{2}^{2} \\
&= f\left(\boldsymbol{w}^{t}\right) - \gamma\eta_t \sum_{k=1}^{K}\left\|\overline{\boldsymbol{g}}_{\boldsymbol{w}_{m_k}}^{t}\right\|_{2}^{2} - \gamma\eta_t \sum_{k=1}^{K}\left(\overline{\boldsymbol{g}}_{\boldsymbol{w}_{m_k}}^{t}\right)^{T}\left(\boldsymbol{g}_{\boldsymbol{w}_{m_k}}^{d_k,t} - \overline{\boldsymbol{g}}_{\boldsymbol{w}_{m_k}}^{t}\right) \\
&+ L(\gamma\eta_t)^2\sum_{k=1}^{K}\left\|\overline{\boldsymbol{g}}_{\boldsymbol{w}_{\boldsymbol{m_k}}}^{t}\right\|_{2}^{2} + L(\gamma\eta_t)^2\sum_{k=1}^{K}\left\|\boldsymbol{g}_{\boldsymbol{w}_{m_k}}^{d_k,t} - \overline{\boldsymbol{g}}_{\boldsymbol{w}_{m_k}}^{t}\right\|_{2}^{2} \\
&+ 2L(\gamma\eta_t)^2\sum_{k=1}^{K}\left(\overline{\boldsymbol{g}}_{\boldsymbol{w}_{m_k}}^{t}\right)^{T}\left(\boldsymbol{g}_{\boldsymbol{w}_{m_k}}^{d_k,t} - \overline{\boldsymbol{g}}_{\boldsymbol{w}_{m_k}}^{t}\right) - \gamma\eta_t \sum_{k=1}^{K}\left(\overline{\boldsymbol{g}}_{\boldsymbol{w}_{m_k}}^{t}\right)^{T}\Delta g_{\boldsymbol{w}_{m_k}}^{t} \\
&+ L(\gamma\eta_t)^2\sum_{k=1}^{K}\left\|\Delta g_{\boldsymbol{w}_{m_k}}^{t}\right\|_{2}^{2} \\
&= f\left(\boldsymbol{w}^{t}\right) - \left(\gamma\eta_t - L(\gamma\eta_t)^2\right)\sum_{k=1}^{K}\left\|\overline{\boldsymbol{g}}_{\boldsymbol{w}_{m_k}}^{t}\right\|_{2}^{2} + \tilde{Q}_1 + \tilde{Q}_2 + \tilde{Q}_3.
\end{aligned} \tag{45}$$

where

$$\tilde{Q}_1 = L(\gamma\eta_t)^2 \sum_{k=1}^{K} \left\| \boldsymbol{g}_{\boldsymbol{w}_{m_k}}^{d_k,t} - \overline{\boldsymbol{g}}_{\boldsymbol{w}_{m_k}}^{t} \right\|_2^2, \tag{46}$$

$$\tilde{Q}_2 = \left(2L(\gamma\eta_t)^2 - \gamma\eta_t\right) \sum_{k=1}^{K} \left(\overline{\boldsymbol{g}}_{\boldsymbol{w}_{m_k}}^{t}\right)^T \left(\boldsymbol{g}_{\boldsymbol{w}_{m_k}}^{d_k,t} - \overline{\boldsymbol{g}}_{\boldsymbol{w}_{m_k}}^{t}\right), \tag{47}$$

$$\tilde{Q}_3 = -\gamma\eta_t \sum_{k=1}^{K} \left(\overline{\boldsymbol{g}}_{\boldsymbol{w}_{m_k}}^{t}\right)^T \Delta g_{\boldsymbol{w}_{m_k}}^{t} + L(\gamma\eta_t)^2 \sum_{k=1}^{K} \left\| \Delta g_{\boldsymbol{w}_{m_k}}^{t} \right\|_2^2. \tag{48}$$

To prove the convergence, we then show that the expectations of $\tilde{Q}_1$, $\tilde{Q}_2$ and $\tilde{Q}_3$ are bounded respectively. The expectation of $\tilde{Q}_1$ is bounded by

$$\begin{aligned}
\mathbb{E}_{\boldsymbol{x}_t}\left[\tilde{Q}_1\right] &= L(\gamma\eta_t)^2 \mathbb{E}_{\boldsymbol{x}_t}\left[\sum_{k=1}^{K} \left\| \boldsymbol{g}_{\boldsymbol{w}_{m_k}}^{d_k,t} - \overline{\boldsymbol{g}}_{\boldsymbol{w}_{m_k}}^{t} \right\|_2^2\right] \\
&= L(\gamma\eta_t)^2 \mathbb{E}_{\boldsymbol{x}_t}\left[\sum_{k=1}^{K} \left\| \boldsymbol{g}_{\boldsymbol{w}_{m_k}}^{d_k,t} - \overline{\boldsymbol{g}}_{\boldsymbol{w}_{m_k}}^{d_k,t} - \overline{\boldsymbol{g}}_{\boldsymbol{w}_{m_k}}^{t} + \overline{\boldsymbol{g}}_{\boldsymbol{w}_{m_k}}^{d_k,t} \right\|_2^2\right] \\
&\leq 2L(\gamma\eta_t)^2 \mathbb{E}_{\boldsymbol{x}_t}\left[\sum_{k=1}^{K} \left\| \boldsymbol{g}_{\boldsymbol{w}_{m_k}}^{d_k,t} - \overline{\boldsymbol{g}}_{\boldsymbol{w}_{m_k}}^{d_k,t} \right\|_2^2\right] + 2L(\gamma\eta_t)^2 \sum_{k=1}^{K} \left\| \overline{\boldsymbol{g}}_{\boldsymbol{w}_{m_k}}^{d_k,t} - \overline{\boldsymbol{g}}_{\boldsymbol{w}_{m_k}}^{t} \right\|_2^2 \\
&= 2L(\gamma\eta_t)^2 \sum_{k=1}^{K} \mathbb{E}_{\boldsymbol{x}_t}\left[\left\| \boldsymbol{g}_{\boldsymbol{w}_{m_k}}^{d_k,t} - \overline{\boldsymbol{g}}_{\boldsymbol{w}_{m_k}}^{d_k,t} \right\|_2^2\right] + 2L(\gamma\eta_t)^2 \sum_{k=1}^{K} \left\| \overline{\boldsymbol{g}}_{\boldsymbol{w}_{m_k}}^{d_k,t} - \overline{\boldsymbol{g}}_{\boldsymbol{w}_{m_k}}^{t} \right\|_2^2 \\
&\leq 2L(\gamma\eta_t)^2 \sum_{k=1}^{K} \mathbb{E}_{\boldsymbol{x}_t}\left[\left\| \boldsymbol{g}_{\boldsymbol{w}_{m_k}}^{d_k,t} \right\|_2^2\right] + 2L(\gamma\eta_t)^2 \sum_{k=1}^{K} \left\| \overline{\boldsymbol{g}}_{\boldsymbol{w}_{m_k}}^{d_k,t} - \overline{\boldsymbol{g}}_{\boldsymbol{w}_{m_k}}^{t} \right\|_2^2 \\
&\leq 2L(\gamma\eta_t)^2 MK + 2L(\gamma\eta_t)^2 \sum_{k=1}^{K} \left\| \overline{\boldsymbol{g}}_{\boldsymbol{w}_{m_k}}^{d_k,t} - \overline{\boldsymbol{g}}_{\boldsymbol{w}_{m_k}}^{t} \right\|_2^2 \\
&= 2L(\gamma\eta_t)^2 MK + 2L(\gamma\eta_t)^2 \tilde{P}_1
\end{aligned} \tag{49}$$

where the first inequality comes from $\|\boldsymbol{x}+\boldsymbol{y}\|_2^2 \leq 2\|\boldsymbol{x}\|_2^2 + 2\|\boldsymbol{y}\|_2^2$. The second inequality is due to $\mathbb{E}\left[\|\epsilon - \mathbb{E}[\epsilon]\|_2^2\right] \leq \mathbb{E}\left[\|\epsilon\|_2^2\right] - \|\mathbb{E}[\epsilon]\|_2^2 \leq \mathbb{E}\left[\|\epsilon\|_2^2\right]$ and $\mathbb{E}_{\boldsymbol{x}_t}\left[\boldsymbol{g}_{\boldsymbol{w}_{m_k}}^{d_k,t}\right] = \overline{\boldsymbol{g}}_{\boldsymbol{w}_{m_k}}^{d_k,t}$. The third inequality follows from Assumption 3. $\tilde{P}_1$ is bounded by

$$
\begin{aligned}
\tilde{P}_1 &= \sum_{k=1}^{K} \left\| \overline{\boldsymbol{g}}_{\boldsymbol{w}_{m_k}}^{d_{k,t}} - \overline{\boldsymbol{g}}_{\boldsymbol{w}_{m_k}}^{t} \right\|_2^2 \leq L^2 \sum_{k=1}^{K} \left\| \boldsymbol{w}_{m_k}^{t} - \boldsymbol{w}_{m_k}^{d_{k,t}} \right\|_2^2 \\
&= L^2 \sum_{k=1}^{K} \left\| \sum_{j=\max\{0,d_{k,t}\}}^{t-1} \left(\boldsymbol{w}_{m_k}^{j+1} - \boldsymbol{w}_{m_k}^{j} \right) \right\|_2^2 \leq L^2 \sum_{k=1}^{K} \sum_{j=\max\{0,d_{k,t}\}}^{t-1} \left\| \boldsymbol{w}_{m_k}^{j+1} - \boldsymbol{w}_{m_k}^{j} \right\|_2^2 \\
&= L^2 \sum_{k=1}^{K} \sum_{j=\max\{0,d_{k,t}\}}^{t-1} (\gamma\eta_j)^2 \left\| \boldsymbol{g}_{\boldsymbol{w}_{m_k}}^{d_{k,t}} \right\|_2^2 \leq L^2 M \sum_{k=1}^{K} \sum_{j=\max\{0,d_{k,t}\}}^{t-1} (\gamma\eta_j)^2 \\
&\leq (\gamma\eta_t)^2 L^2 M \sum_{k=1}^{K} (t - \max\{0, d_{k,t}\}) \leq (\gamma\eta_t)^2 L^2 M \sum_{k=1}^{K} 2(K-k)
\end{aligned} \tag{50}
$$

where the first inequality follows from Assumption 2. The expectation of $\tilde{Q}_2$ is bounded by

$$
\begin{aligned}
\mathbb{E}_{\boldsymbol{x}_t}\left[\tilde{Q}_2\right] &= -\left(\gamma\eta_t - 2L(\gamma\eta_t)^2\right) \mathbb{E}_{\boldsymbol{x}_t}\left[\sum_{k=1}^{K} \left(\overline{\boldsymbol{g}}_{\boldsymbol{w}_{m_k}}^{t} \right)^T \left(\boldsymbol{g}_{\boldsymbol{w}_{m_k}}^{d_{k,t}} - \overline{\boldsymbol{g}}_{\boldsymbol{w}_{m_k}}^{t} \right) \right] \\
&= -\left(\gamma\eta_t - 2L(\gamma\eta_t)^2\right) \sum_{k=1}^{K} \left(\overline{\boldsymbol{g}}_{\boldsymbol{w}_{m_k}}^{t} \right)^T \left(\overline{\boldsymbol{g}}_{\boldsymbol{w}_{m_k}}^{d_{k,t}} - \overline{\boldsymbol{g}}_{\boldsymbol{w}_{m_k}}^{t} \right) \\
&\leq \frac{\gamma\eta_t - 2L(\gamma\eta_t)^2}{2} \sum_{k=1}^{K} \left\| \overline{\boldsymbol{g}}_{\boldsymbol{w}_{m_k}}^{t} \right\|_2^2 + \frac{\gamma\eta_t - 2L(\gamma\eta_t)^2}{2} \tilde{P}_1
\end{aligned} \tag{51}
$$

where the equality follows from $\pm \boldsymbol{x}^T \boldsymbol{y} \leq \frac{1}{2}\|\boldsymbol{x}\|_2^2 + \frac{1}{2}\|\boldsymbol{y}\|_2^2$ and the second term on the right hand side is in the form of $\tilde{P}_1$ whose bound has been proved. The expectation of $\tilde{Q}_3$ is bounded by

$$
\begin{aligned}
\mathbb{E}_{\boldsymbol{x}_t}\left[\tilde{Q}_3\right] &= -\gamma\eta_t \sum_{k=1}^{K} \mathbb{E}_{\boldsymbol{x}_t}\left[\left(\overline{\boldsymbol{g}}_{\boldsymbol{w}_{m_k}}^{t} \right)^T \Delta g_{\boldsymbol{w}_{m_k}}^{t} \right] + L(\gamma\eta_t)^2 \sum_{k=1}^{K} \mathbb{E}_{\boldsymbol{x}_t}\left[\left\| \Delta g_{\boldsymbol{w}_{m_k}}^{t} \right\|_2^2 \right] \\
&= \gamma\eta_t \sum_{k=1}^{K} \left(\overline{\boldsymbol{g}}_{\boldsymbol{w}_{m_k}}^{t} \right)^T \mathbb{E}_{\boldsymbol{x}_t}\left[\Delta g_{\boldsymbol{w}_{m_k}}^{t} \right] + L(\gamma\eta_t)^2 \sum_{k=1}^{K} \mathbb{E}_{\boldsymbol{x}_t}\left[\left\| \Delta g_{\boldsymbol{w}_{m_k}}^{t} \right\|_2^2 \right] \\
&\leq L(\gamma\eta_t)^2 M_g K
\end{aligned} \tag{52}
$$

where the last inequality comes from Assumption 1. Taking the expectation of both sides in Eq. (45) and substituting $\tilde{Q}_1$, $\tilde{Q}_2$ and $\tilde{Q}_3$, it can be rewritten as

$$
\begin{aligned}
\mathbb{E}_{\boldsymbol{x}_t}\left[f\left(\boldsymbol{w}^{t+1}\right)\right] &\leq f\left(\boldsymbol{w}^{t}\right) - \left((\gamma\eta_t) - L(\gamma\eta_t)^2\right)\sum_{k=1}^{K}\left\|\overline{\boldsymbol{g}}^{t}_{\boldsymbol{w}_{m_k}}\right\|_2^2 \\
&+ 2L(\gamma\eta_t)^2 MK + 2L(\gamma\eta_t)^2\tilde{P}_1 + \frac{\gamma\eta_t - 2L(\gamma\eta_t)^2}{2}\sum_{k=1}^{K}\left\|\overline{\boldsymbol{g}}^{t}_{\boldsymbol{w}_{m_k}}\right\|_2^2 \\
&+ \frac{\gamma\eta_t - 2L(\gamma\eta_t)^2}{2}\tilde{P}_1 + L(\gamma\eta_t)^2 M_g K \\
&\leq f\left(\boldsymbol{w}^{t}\right) - \gamma\eta_t\sum_{k=1}^{K}\left\|\overline{\boldsymbol{g}}^{t}_{\boldsymbol{w}_{m_k}}\right\|_2^2 \\
&+ \frac{(\gamma\eta_t) + 2L(\gamma\eta_t)^2}{2}(\gamma\eta_t)^2 L^2 M\sum_{k=1}^{K}2(K-k) + L(\gamma\eta_t)^2(2M+M_g)K \\
&= f\left(\boldsymbol{w}^{t}\right) - \gamma\eta_t\sum_{k=1}^{K}\left\|\overline{\boldsymbol{g}}^{t}_{\boldsymbol{w}_{m_k}}\right\|_2^2 \\
&+ (\gamma\eta_t)^2\left(L(2M+M_g)K + \frac{\gamma\eta_t + 2L(\gamma\eta_t)^2}{2}L^2 M\sum_{k=1}^{K}2(K-k)\right) \\
&\leq f\left(\boldsymbol{w}^{t}\right) - \gamma\eta_t\sum_{k=1}^{K}\left\|\overline{\boldsymbol{g}}^{t}_{\boldsymbol{w}_{m_k}}\right\|_2^2 \\
&+ (\gamma\eta_t)^2\left(L(2M+M_g)K + LM\sum_{k=1}^{K}3(K-k)\right)
\end{aligned} \tag{53}
$$

where the last inequality follows from $L\gamma\eta_t \leq 1$ such that

$$
\frac{\gamma\eta_t + 2L(\gamma\eta_t)^2}{2}L = \frac{L\gamma\eta_t}{2} + (L\gamma\eta_t)^2 \leq \frac{3}{2}. \tag{54}
$$

The proof is now completed.

S2 Appendix

Proof of Theorem 2. From Theorem 1, we obtain that

$$
\begin{aligned}
\gamma\eta_t\sum_{k=1}^{K}\left\|\overline{\boldsymbol{g}}^{t}_{\boldsymbol{w}_{m_k}}\right\|_2^2 &\leq f\left(\boldsymbol{w}^{t}\right) - \mathbb{E}_{\boldsymbol{x}_t}\left[f\left(\boldsymbol{w}^{t+1}\right)\right] \\
&+ (\gamma\eta_t)^2\left(L(2M+M_g)K + LM\sum_{k=1}^{K}3(K-k)\right)
\end{aligned} \tag{55}
$$

$$
\begin{aligned}
\gamma\eta_t\left\|\overline{\boldsymbol{g}}^{t}_{\boldsymbol{w}}\right\|_2^2 &\leq f\left(\boldsymbol{w}^{t}\right) - \mathbb{E}_{\boldsymbol{x}_t}\left[f\left(\boldsymbol{w}^{t+1}\right)\right] \\
&+ (\gamma\eta_t)^2\left(L(2M+M_g)K + LM\sum_{k=1}^{K}3(K-k)\right).
\end{aligned} \tag{56}
$$

Then we take the expectation of both sides

$$
\begin{aligned}
\gamma\eta_t\mathbb{E}[\left\|\overline{\boldsymbol{g}}^{t}_{\boldsymbol{w}}\right\|_2^2] &\leq \mathbb{E}[f\left(\boldsymbol{w}^{t}\right)] - \mathbb{E}\left[f\left(\boldsymbol{w}^{t+1}\right)\right] \\
&+ (\gamma\eta_t)^2\left(L(2M+M_g)K + LM\sum_{k=1}^{K}3(K-k)\right).
\end{aligned} \tag{57}
$$

By summing both sides of above inequality from 0 to $T-1$, and dividing it by $\mathcal{T}_T = \sum_{t=0}^{T-1} \gamma\eta_t$, it becomes

$$
\begin{aligned}
\frac{1}{\mathcal{T}_T}\sum_{t=0}^{T-1}\gamma\eta_t\mathbb{E}\left\|\bar{\boldsymbol{g}}_{\boldsymbol{w}}^t\right\|_2^2 \leq\ & \frac{f\left(\boldsymbol{w}^0\right)-\mathbb{E}\left[f\left(\boldsymbol{w}^T\right)\right]}{\mathcal{T}_T} \\
&+\frac{\left(L(2M+M_g)K+LM\sum_{k=1}^{K}3(K-k)\right)\sum_{t=0}^{T-1}\gamma\eta_t^2}{\mathcal{T}_T} \\
\leq\ & \frac{f\left(\boldsymbol{w}^0\right)-f\left(\boldsymbol{w}^*\right)}{\mathcal{T}_T} \\
&+\frac{\left(L(2M+M_g)K+LM\sum_{k=1}^{K}3(K-k)\right)\sum_{t=0}^{T-1}\gamma\eta_t^2}{\mathcal{T}_T}.
\end{aligned}
\tag{58}
$$

where the last inequality comes from $f\left(\boldsymbol{w}^*\right) \leq \mathbb{E}\left[f\left(\boldsymbol{w}^T\right)\right]$.

S3 Appendix

Proof of Theorem 3. Setting the learning rate in Eq. (57) to be a constant (i.e., $\eta_t = \eta$), Eq. (57) is rewritten as

$$
\begin{aligned}
\mathbb{E}\left[\left\|\bar{\boldsymbol{g}}_{\boldsymbol{w}}^t\right\|_2^2\right] \leq\ & \frac{\mathbb{E}\left[f\left(\boldsymbol{w}^t\right)\right]-\mathbb{E}\left[f\left(\boldsymbol{w}^{t+1}\right)\right]}{\gamma\eta} \\
&+(\gamma\eta)\left(L(2M+M_g)K+LM\sum_{k=1}^{K}3(K-k)\right).
\end{aligned}
\tag{59}
$$

Summing both sides of Eq. (59) from $t=0$ to $t=T-1$ and dividing them by T, it results in

$$
\begin{aligned}
\frac{1}{T}\sum_{t=0}^{T-1}\mathbb{E}\left[\left\|\bar{\boldsymbol{g}}_{\boldsymbol{w}}^t\right\|_2^2\right] \leq\ & \frac{f\left(\boldsymbol{w}^0\right)-\mathbb{E}\left[f\left(\boldsymbol{w}^T\right)\right]}{\gamma\eta T} \\
&+(\gamma\eta)\left(L(2M+M_g)K+LM\sum_{k=1}^{K}3(K-k)\right) \\
\leq\ & \frac{f\left(\boldsymbol{w}^0\right)-f\left(\boldsymbol{w}^*\right)}{\gamma\eta T}+(\gamma\eta)\left(L(2M+M_g)K+LM\sum_{k=1}^{K}3(K-k)\right) \\
=\ & \frac{f\left(\boldsymbol{w}^0\right)-f\left(\boldsymbol{w}^*\right)+(\gamma\eta)^2T\left(L(2M+M_g)K+LM\sum_{k=1}^{K}3(K-k)\right)}{\gamma\eta T}.
\end{aligned}
\tag{60}
$$

Substituting $\gamma\eta = \epsilon\sqrt{\frac{\left(f\left(\boldsymbol{w}^0\right)-f\left(\boldsymbol{w}^*\right)\right)}{T\left(L(2M+M_g)K+LM\sum_{k=1}^{K}3(K-k)\right)}}$ into Eq. (60), the right hand side becomes

$$
\begin{aligned}
&\frac{\left(1+\epsilon^2\right)\left(f\left(\boldsymbol{w}^0\right)-f\left(\boldsymbol{w}^*\right)\right)}{T\epsilon\sqrt{\frac{\left(f\left(\boldsymbol{w}^0\right)-f\left(\boldsymbol{w}^*\right)\right)}{T\left(L(2M+M_g)K+LM\sum_{k=1}^{K}3(K-k)\right)}}} \\
&=\frac{\left(1+\epsilon^2\right)}{\epsilon}\sqrt{\frac{\left(f\left(\boldsymbol{w}^0\right)-f\left(\boldsymbol{w}^*\right)\right)\left(L(2M+M_g)K+LM\sum_{k=1}^{K}3(K-k)\right)}{T}}.
\end{aligned}
\tag{61}
$$

While $\frac{1}{T}\sum_{t=0}^{T-1}\mathbb{E}\left[\left\|\overline{\boldsymbol{g}}_{\boldsymbol{w}}^{t}\right\|_2^2\right]$ is the average of $\mathbb{E}\left[\left\|\overline{\boldsymbol{g}}_{\boldsymbol{w}}^{t}\right\|_2^2\right]$ for $t = 0, 1, \ldots, T-1$, we have

$$\min_{t\in\{0,1,\ldots,T-1\}}\mathbb{E}\left[\left\|\overline{\boldsymbol{g}}_{\boldsymbol{w}}^{t}\right\|_2^2\right] \leq \frac{1}{T}\sum_{t=0}^{T-1}\mathbb{E}\left[\left\|\overline{\boldsymbol{g}}_{\boldsymbol{w}}^{t}\right\|_2^2\right] \leq \frac{\left(1+\epsilon^2\right)}{\epsilon}\sqrt{\frac{\left(f\left(\boldsymbol{w}^0\right)-f\left(\boldsymbol{w}^*\right)\right)\left(L(2M+M_g)K+LM\sum_{k=1}^{K}3(K-k)\right)}{T}} \tag{62}$$

which completes the proof.
